# Evidence of focusing the MHC class I immunopeptidome by tapasin

**DOI:** 10.3389/fimmu.2025.1563789

**Published:** 2025-05-08

**Authors:** Rachel Darley, Patricia T. Illing, Patrick Duriez, Alistair Bailey, Anthony W. Purcell, Andy van Hateren, Tim Elliott

**Affiliations:** ^1^ Institute for Life Sciences and Centre for Cancer Immunology, Faculty of Medicine, University of Southampton, Southampton, United Kingdom; ^2^ Department of Biochemistry and Molecular Biology and Biomedicine Discovery Institute, Monash University, Melbourne, VIC, Australia; ^3^ Cancer Research UK Protein Core Facility, Faculty of Medicine, University of Southampton, Southampton, United Kingdom; ^4^ Centre for Immuno-Oncology and Chinese Academy of Medical Sciences (CAMS)-Oxford Institute, Nuffield Department of Medicine, University of Oxford, Oxford, United Kingdom

**Keywords:** MHC class I, tapasin, TAPBPR, peptide editing, peptide selection, immunopeptidome

## Abstract

Major Histocompatibility Complex class I (MHC-I) molecules bind and present peptides to cytotoxic T cells, protecting against pathogens and cancer. MHC-I is highly polymorphic and each allotype is promiscuous, and capable of binding a unique and diverse repertoire of peptide ligands. The peptide editing chaperone tapasin optimizes this allotype specific repertoire of peptides, resulting in the selection of high affinity peptides. MHC-I allotypes differ in the extent they engage tapasin. This suggests that tapasin-dependent MHC-I allotypes should present a less diverse repertoire that is enriched in higher-affinity peptides, and which are present in higher abundance, than tapasin independent MHC-I allotypes, which should present a broader repertoire containing peptides with a lower average affinity. Experimental verification of this hypothesis has been confounded by the different peptide binding specificities of MHC-I allotypes. Here, we independently investigated the peptide focusing function of tapasin by introducing a point mutation into a tapasin independent MHC-I allotype that dramatically increased its tapasin dependence without substantially altering its peptide binding specificity. This allowed us to demonstrate ligand focusing by tapasin at both the repertoire level *in cellulo*, and by using an *in vitro* system in which tapasin was artificially tethered to MHC-I, at the individual peptide level. We found that tapasin had a greater influence on tapasin dependent MHC-I molecules, and that tapasin modulated peptide selection according to peptide-MHC-I complex stability, disfavoring short-lived peptide-MHC-I complexes. Thus, tapasin dependent MHC-I molecules experience greater tapasin filtering, resulting in less diverse MHC-I immunopeptidomes that are enriched in high affinity peptide-MHC-I complexes.

## 1 Introduction

Major Histocompatibility Complex class I (MHC-I) molecules are an important component of the adaptive immune response and provide protection from pathogens and cancer by binding intracellular peptides and presenting them at the cell surface to specialized immune cells including cytotoxic T cells. The peptides presented by MHC-I molecules (the MHC-I immunopeptidome) are predominantly selected from a diverse pool of peptides transported into the endoplasmic reticulum following proteasome mediated degradation of intracellular proteins and defective nascent polypeptides, which can be further refined by aminopeptidases within the ER.

MHC-I peptide selection is orchestrated by peptide loading complexes (PLC), which are centered upon TAP peptide transporters and include a number of chaperones: tapasin; calreticulin; and ERp57, and which synergistically co-ordinate recruitment of nascent MHC-I molecules and their peptide loading (1). Of the PLC constituents, tapasin assists MHC-I to preferentially select high affinity peptides for presentation, forming stable, long-lived peptide-MHC-I complexes (2–5). Once loaded with peptide, MHC-I complexes are released from the PLC and exit the ER, where some MHC-I allotypes encounter further scrutiny from the tapasin homologue TAPBPR (6). Like tapasin, TAPBPR refines the peptides presented by MHC-I, preferentially favoring high affinity peptides (7, 8). Empty or sub-optimally loaded MHC-I (i.e. those MHC-I molecules containing low affinity binding peptides) may also be returned to the ER following retrieval from the endoplasmic-reticulum–Golgi intermediate compartment in a calreticulin dependent manner (9–11).

MHC-I molecules are highly polymorphic, with MHC-I allotypes binding different repertoires of peptides depending on the molecular composition of their peptide-binding grooves (12, 13). In addition, MHC-I allotypes differ in their dependence upon tapasin for the selection of peptide cargoes that permit stable cell surface expression (2, 14–16). While all MHC-I allotypes benefit from tapasin to some extent, this ranges from allotypes such as HLA-B*44:02 for which tapasin is essentially obligatory for peptide selection and cell surface expression, to allotypes such as HLA-B*44:05 which can efficiently select and present a repertoire of peptides at the cell surface in the absence of tapasin (2, 14, 15, 17). Additionally, TAPBPR has specificity for a select group of MHC-I allotypes, with a strong preference for some HLA-A gene products (6, 18, 19).

Tapasin and TAPBPR mediated peptide editing therefore underpins the diversity of the MHC-I immunopeptidome that is presented to T cells and is an important factor in determining the breadth of an immune response and protection against potentially lethal infections and cancer (14, 20, 21). Experimental and computational studies have suggested that the diversity of peptides presented by different human MHC-I allotypes varies (22–26). In humans, the cell surface expression levels of four MHC-I allotypes was shown to be inversely correlated with the breadth of their immunopeptidomes (24, 25, 27). Interestingly, Chappell et al. noted that the cell surface expression levels of these allotypes correlated with their tapasin dependence (15, 27). Indeed, the tapasin dependence of a wide variety of HLA-A and HLA-B allotypes was measured and shown to be inversely correlated with the number of peptides derived from HIV that elicited an immunogenic response (14). Collectively, these studies suggest that tapasin dependent MHC-I allotypes may present a less diverse range of peptides, which is enriched in high affinity peptides and present at higher surface expression levels, compared with tapasin independent MHC-I allotypes, and that these factors are important determinants for successful immune responses. Similarly, a more diverse repertoire of peptides was identified in TAPBPR depleted cells compared with TAPBPR expressing cells, suggesting that TAPBPR restricts the diversity of peptides presented by some MHC-I molecules (7). However, tapasin or TAPBPR mediated focusing of MHC-I peptide repertoires has not been formally demonstrated, mostly because of the difficulty of distinguishing between whether the differential peptide focusing experienced by MHC-I allotypes is a consequence of variation in their ability to bind tapasin, or TAPBPR, and exploit their peptide editing potential, or because of differences in peptide selectivity imposed by the composition of the peptide binding grooves.

We recently demonstrated the relationship between tapasin activity and repertoire focusing by tuning the level of tapasin expression and measuring changes in peptide editing intensity (28). Here, we build upon these findings by measuring the influence that tapasin dependence has on the composition of immunopeptidomes selected by highly similar human MHC-I molecules. We next characterized how tapasin modulates MHC-I peptide selection and the magnitude of tapasin optimization experienced by MHC-I allotypes using an *in vitro* system.

## 2 Materials and methods

### 2.1 Analysis of HLA-B*44:02, HLA-B*44:05 and HLA-B*44:05-W147A immunopeptidomes

Plasmids encoding full length HLA-B*44:02, HLA-B*44:05 and HLA-B*44:05-W147A proteins, and the generation of stable transfectants of 721.220 cells reconstituted with human tapasin have been described previously (29). Approximately 5x10^8^ cells were grown for each cell line, and cell pellets were snap frozen. Cell lysis and MHC-I peptide isolation were performed as previously described in detail (30). Briefly, cell pellets were lysed by cryomilling and incubation in a non-denaturing lysis buffer (0.5% Igepal CA-630, 50 mM Tris pH 8.0, 150 mM NaCl, 1 x Roche Complete protease inhibitor cocktail). MHC-I complexes were captured from the lysate using 7.5 mg of anti-MHC-I antibody, W6/32, immobilized on protein A Sepharose and subsequently dissociated with 10% acetic acid. The peptides were fractionated and separated from β_2_m and heavy chain components by reversed-phase high performance liquid chromatography (RP-HPLC). The peptide containing fractions were concentrated and chromatographically distant fractions were combined to generate nine pools, reconstituted in 15 µL 0.1% formic acid containing 500 fmol iRT reference peptides (31). Pools were analyzed by liquid chromatography-tandem mass spectrometry (LC-MS/MS) using a SCIEX 5600+ mass spectrometer, equipped with a NanoUltra cHiPLC system (Eksigent) and a Nanospray III ion source. Peptides were trapped on a cHiPLC trap column (3 µm, ChromXP C18CL, 120 Å, 0.5 mm x 200 µm), by loading at 5 µL/min in 0.1% formic acid, 2% acetonitrile, prior to elution over a cHiPLC column (3 µm, ChromXP C18CL, 120 Å, 15 cm x 75 µm) at 300 nL/min with increasing acetonitrile over 75 minutes. The mass spectrometer was operated as follows: ion spray voltage 2400 V, curtain gas 25 l/min, ion source gas 20 l/min, interface heater temperature 150°C, MS1 range 200-1800, MS1 accumulation time 200 ms, MS2 range 60-1800, and MS2 accumulation time 150 ms. The top 20 ions meeting the following criteria were selected for MS/MS fragmentation with rolling collision energy: >200 Da, charge state +2 to +5, >40 cps, dynamic exclusion 30 seconds after two occurrences. Mass spectra were interpreted by database search against the Uniprot/SwissProt (32) reviewed human proteome accessed October 2018 using PEAKS Studio X_PRO_ (10.6 build, Bioinformatics Solutions Inc.) and a contaminant database contain the iRT reference peptide sequences using the following parameters: Instrument Triple TOF, fragmentation CID, parent mass error tolerance 25ppm, fragment mass error tolerance 0.1Da, enzyme none, digest mode unspecific, variable modifications Oxidation (M) +15.99 and Deamidation (NQ) +0.98, max variable modifications per peptide 3, False discovery rate (FDR) estimation enabled. A 5% peptide FDR was applied. Peptides identified from similar isolations of MHC class II from closely related 721.221 derived cell lines were excluded from downstream analyses. Analyses were performed using peptides of 8–13 amino acids, non-redundant by sequence (i.e. modifications were not considered), consistent with MHC-I ligands.

Peptide motifs were assigned via MixMHCp (v2.1, ref (33)) with two motifs specified for HLA-B*44:02 as described in Ref (34). Peptide affinities were predicted using NetMHCpan 4.1 (35), and were represented in nM values, with HLA-B*44:05 specified as the reference HLA supertype for HLA-B*44:05-W147A. Gibbs clustering was performed using Gibbs Cluster 2.0 using pre-set parameters for MHC class I ligands of length 8-13 (36).

### 2.2 Data availability

Mass spectrometry proteomics data have been deposited in the ProteomeXchange Consortium via the PRIDE (37) partner repository under the accession code PXD054743.

### 2.3 Synthetic peptides

The following peptides were used in *in vitro* peptide competition experiments:

HLA-B*44:05 and HLA-B*44:05-W147A: SEIKETNDTW, AETYVEGQRI, YEGQFKDNMF, AEDELAMRGF, EEVEQGVKF, SEIVGSIKM, EEGPSSVRF, SEDEGNLRF, SEMKVSSTW, SEMKVSSTWL, QEMPWNVRM, HESGASIKI, AENEELHQLW, TEVLSNVKF, YEHEKDLVW, DENPQQLKL, KEQNYSDDVL, LESDSFLKF, AELESVLSHL, LEAEADKIGL, AEFAKLVEEF, AELFMEQQHL, AETEGILQKL, EEFGKAFSF. Apart from EEFGKAFSF, the HLA-B*44:05 and HLA-B*44:05-W147A peptides used in the peptide competition experiments were selected from the analysis of the immunopeptidomes or from the Immune Epitope Database (38).

HLA-B*35:01 and HLA-B*35:03: YPLHEQHGM, APLHEQHGM, YALHEQHGM, YPAHEQHGM, YPLKEQHGM, YPLHAQHGM, YPLHEAHGM, YPLHEQAGM, YPLHEQHAM, YPLHEQHGA, LPSSADVEF, LPSKADVEF, FPSDSWCYF, FPSDSWAYF, QFADVIVLF, FPFKYAAAF, KPIVVLHGY, LPPLDITPY, LPPAWQPFL, TPERMAEAGF, CPTENEPDL, CPTENEPDY, EPDLAQCFF, EPDLAQCFY. The HLA-B*35:01 and HLA-B*35:03 peptides used in the peptide competition experiments were selected from references (39–46).

HLA-A*02:01: YLENGKETL, YLVAEKVTV, KLWEAESKL, KLVKEVIAV, GLDDIKDLKV, FLLAEDTKV, SLLENLEKI, FLFEPVVKA, YVVPFVAKV, FLPSDCFPSV, NLVPMVATV, NAVPMVATV, IYSYMDDLYV, IYQYMDDLYV, ICQYMDDLYV, YQYMDDLYV, VLIGPTPVNII, VLVGPTPVNI, VLVGPTPINI, VLIGPTPVNI. The HLA-A*02:01 peptides used in the peptide competition experiments were selected from references (7, 47).

The fluorescent tetramethylrhodamine (TAMRA) labelled peptides: EEFGK^TAMRA^AFSF and YPLK^TAMRA^EQHGM, where K^TAMRA^ denotes TAMRA labelled lysine and the unlabeled competing peptides were synthesized by Syn Peptides (Shanghai, China). The following UV-labile conditional peptide ligands were utilized: SEIDTVAjY, KILGFVFjV, KPIVVLjGY and LPSSADjEF, where j represents 3-amino-3-(2-nitro)phenyl-propionic acid. The UV conditional peptides and the TAMRA labelled FLPSDC^TAMRA^FPSV peptide, where the side chain of cysteine was labelled with 5-TAMRA-maleimide, were synthesized by Peptide Synthetics (Fareham UK). All peptides were reconstituted in dimethyl sulfoxide (DMSO).

### 2.4 Production of peptide-receptive MHC-I molecules

Plasmids encoding human β_2_-microglobulin and HLA-A*02:01-fos have been described previously (7). Nucleotides encoding amino acids 1 to 275 of the mature HLA-B*44:05, HLA-B*44:05-W147A, HLA-B*35:01 and HLA-B*35:03 allotypes were amplified with primers 5’-ATACATATGGGCTCCCACTCCATGA-3’ and 5’-GGAACCTCCCTCCCATCTCAGGGTGAG-3’ from DNA encoding HLA-B*44:05 (2), HLA-B*44:05 W147A (29), HLA-B*35:01 and HLA-B*35:03 (originally kindly supplied by Prof. Raghavan and described in Ref (15), and which had been subsequently sub-cloned into pCDNA3.1). Nucleotides encoding the fos leucine zipper were amplified with primers 5’-AGATGGGAGGGAGGTTCC-3’ and 5’-CGCAAGCTTTTAATGGGC-3’ from DNA encoding HLA-A*02:01-fos. The purified products from the MHC-I and fos PCR reactions were used in a third reaction using primers 5’-ATACATATGGGCTCCCACTCCATGA-3’ and 5’-CGCAAGCTTTTAATGGGC-3’ to create constructs encoding the MHC-I-fos allotypes. Following agarose gel electrophoresis and digestion of the purified products with restriction enzymes the MHC-I-fos sequences were cloned into pET22b (Invitrogen). The fos leucine zipper sequences were removed from HLA-B*44:05-fos and HLA-B*44:05-W147A-fos by amplifying with primers 5’-ATACATATGGGCTCCCACTCCATGA-3’ and 5’-GCCAAGCTTCTACTCCCATCTCAGG-3’ to create HLA-B*44:05 and HLA-B*44:05-W147A constructs without the fos leucine zipper. Following agarose gel electrophoresis and digestion of the purified products with restriction enzymes the HLA-B*44:05 and HLA-B*44:05-W147A sequences were cloned into pET22b (Invitrogen).

Peptide loaded MHC-I complexes were obtained by combining solubilized heavy chain inclusion bodies with solubilized human β_2_m inclusion bodies and the appropriate UV labile peptide: HLA-B*44:05, HLA-B*44:05-fos, HLA-B*44:05-W147A and HLA-B*44:05-W147A-fos: SEIDTVAjY; HLA-B*35:01-fos: KPIVVLjGY; HLA-B*35:03-fos: LPSSADjEF; HLA-A*02:01-fos: KILGFVFjV in 8 M urea, 50 mM MES pH 6.5, 0.1 mM EDTA. Refolding was initiated by 14-fold dilution with cold 100 mM Tris pH 8, 2 mM EDTA, 0.4 M l-arginine hydrochloride, 5 mM reduced glutathione and 0.5 mM oxidized glutathione added over three hours whilst stirring to achieve final concentrations of 1 µM heavy chain, 2 µM β_2_-microglobulin and either 10 µM (HLA-A*02:01), 30 µM (HLA-B*35:03) or 40 µM (HLA-B*35:01, HLA-B*44:05 and HLA-B*44:05-W147A) peptide. Two days later, the protein mixture was concentrated and purified by size exclusion chromatography using a Superdex 200 packed 26/600 gel filtration column (Cytiva) and phosphate buffered saline.

### 2.5 Production of conjugated tapasin-jun-ERp57 C60A proteins and TAPBPR proteins

Plasmids encoding human tapasin-jun with a twin strep affinity purification tag, and ERp57 containing the C60A mutation (48), and the purification of tapasin-jun-ERp57 C60A conjugates, have been described before (49).

Nucleotides encoding amino acids 22 to 406 of human TAPBPR and a His6 affinity purification tag were amplified by PCR using primers 5’-AGCGCGTCTCCAATGAAGCCCCACCCAGCAGAG–3’ and 5’-AGCGCGTCTCCTCCCTTAGTGATGGTGATGGTGGTG–3’ and a plasmid encoding human TAPBPR with a His6 tag kindly supplied by Prof. Louise Boyle (50), and subcloned after the BM40 signal peptide of pDSG102 vector (IBA Life Technologies). DNA encoding human TAPBPR with His6 tag (hTAPBPR-His6) was transfected into MEXI29E cells (IBA Life Technologies) and transfectants were grown in culture for seven days. The cell suspension was centrifuged at 4,000 rpm for 60 minutes and the supernatant passed through a 0.2 µm filter. hTAPBPR-His6 protein was then purified using a 5 ml Nickel Excel column (Cytiva) and equilibration (20 mM sodium phosphate, 0.5 M NaCl, pH 7.4) and elution buffers (20 mM sodium phosphate, 0.5 M NaCL, 500 mM imidazole, pH 7.4). The eluted material was concentrated using 10 k Da spin concentrator columns (Amicon) and further purified using a Superdex 200 packed 26/600 gel filtration column (Cytiva) and 20 mM sodium phosphate, 100 mM NaCl, pH 7.2. The protein was concentrated to around 4 mg/ml, aliquoted and frozen.

### 2.6 *In vitro* peptide competition experiments

Peptide competition experiments were prepared in PBS supplemented with 0.5 mg/ml bovine serum albumin (BSA) and a final concentration of 1.67% DMSO. The indicated concentrations of MHC-I molecules were supplemented with 20x excess β_2_-microglobulin and exposed to 366 nm light for 20 minutes at 4°C. Twenty µl of the UV exposed proteins were added to a 96 well microplate, with each well containing 40 µl of a titration (0 - 83.33 µM) of an unlabeled peptide competitor, 2–3 nM of the appropriate TAMRA labelled peptide, and 300 nM tapasin-jun-ERp57 (HLA-B*44 and HLA-B*35 experiments), or 300 nM TAPBPR (HLA-A*02:01 experiments) or neither tapasin-jun-ERp57 or TAPBPR. Samples were prepared in duplicate and incubated overnight at 25°C.

HLA-B*44:05-fos and HLA-B*44:05-W147A-fos proteins were used at 375 nM with 2 nM EEFGK*AFSF peptide. HLA-B*44:05 and HLA-B*44:05-W147A proteins were used at 160 nM with 2 nM EEFGK*AFSF peptide. HLA-B*35:01-fos and HLA-B*35:03-fos proteins were used at 225 nM with 3 nM YPLK*EQHGM peptide. HLA-A*02:01-fos protein was used at 50 nM with 2 nM FLPSDC*FPSV peptide.

Fluorescence polarization measurements were taken using an I3x (Molecular Devices) with rhodamine detection cartridge. Binding of TAMRA-labelled peptide is reported in milli polarization units (mP) and is obtained from the equation: mP = 1000 x (S – G x P)/(S + G x P), where S and P are background subtracted fluorescence count rates (S = polarization emission filter is parallel to the excitation filter; P = polarization emission filter is perpendicular to the excitation filter and G (grating) is an instrument and assay dependent factor. IC50 values were calculated by performing non-linear regression in GraphPad Prism using the one phase decay model, with plateaus constrained to 50. Apart from the experiments involving the HLA-B*44:05 and HLA-B*44:05-W147A proteins, each peptide was tested in at least two independent experiments, with the mean IC50 values being taken from the replicate experiments.

### 2.7 *In vitro* indirect measurements of peptide-MHC-I complex half-lives

Indirect peptide dissociation experiments were conducted essentially as described in ref (51). Experiments were performed in PBS supplemented with 0.5 mg/ml BSA and a final concentration of 1.67% DMSO. The indicated concentrations of MHC-I molecules were supplemented with 20x excess β_2_-microglobulin and exposed to 366 nm light for 20 minutes at 4°C, before being incubated with an equimolar concentration of each of the unlabeled peptides, or no peptide (no peptide control), overnight at 25°C in a volume of 105.6 µl. The next day 48 µl was added to each well of a 96 well microplate, before 12 µl of the appropriate TAMRA labelled peptide was added to each well, and fluorescence polarization measurements were periodically taken at 25°C for ~200 hours. Samples were prepared in duplicate. Each peptide was tested in at least two independent experiments.

HLA-B*44:05-fos and HLA-B*44:05-W147A-fos proteins were used at 375 nM with 4 nM EEFGK*AFSF. HLA-B*35:01-fos and HLA-B*35:03-fos proteins were used at 225 nM with 3 nM YPLK*EQHGM peptide. HLA-A*02:01-fos protein was used at 50 nM with 2 nM FLPSDC*FPSV peptide.

Peptide-MHC-I half-lives were calculated by performing non-linear regression in GraphPad Prism using the one phase association model, with plateaus constrained to the maximum polarization that was measured in the no peptide control.

## 3 Results

### 3.1 Tapasin skews the immunopeptidome selected by tapasin dependent MHC-I molecules in favor of high affinity peptides

The HLA-B*44:02 and HLA-B*44:05 MHC-I allotypes have been frequently used to investigate tapasin function as they differ by just one amino acid residue (HLA-B*44:02: Asp116, HLA-B*44:05: Tyr116). They share similar peptide binding preferences (17, 26) and are both similarly poor substrates for TAPBPR (18), but vary drastically in tapasin dependence (2, 14, 15, 17, 29). We have found that *in vivo*, the W147A mutation increases the tapasin dependence of the otherwise highly tapasin independent HLA-B*44:05 molecules (**Supplementary Figures 1b, c, e** and ref (29)). Thus, tapasin enhances peptide loading of W147A molecules to a level that is intermediate between highly tapasin dependent HLA-B*44:02 and highly tapasin independent HLA-B*44:05 molecules. Importantly, this mutation did not substantially change peptide binding specificity as assessed by peptide stabilization assays, where five known HLA-B*44 binding peptides increased the recovery of radiolabeled W147A molecules to an approximately similar extent as HLA-B*44:02 and HLA-B*44:05 molecules (**Supplementary Figure 2**). Therefore, the W147A mutant allowed us to investigate the effect of tapasin on the peptide presentation profile of the HLA-B*44:05 molecule in relative isolation of differences in peptide binding specificities. We therefore sought to compare the immunopeptidomes selected by HLA-B*44:02, HLA-B*44:05 and HLA-B*44:05-W147A molecules that experience differential benefit from tapasin, using monoallelic antigen presenting cells.

**Figure 1:**
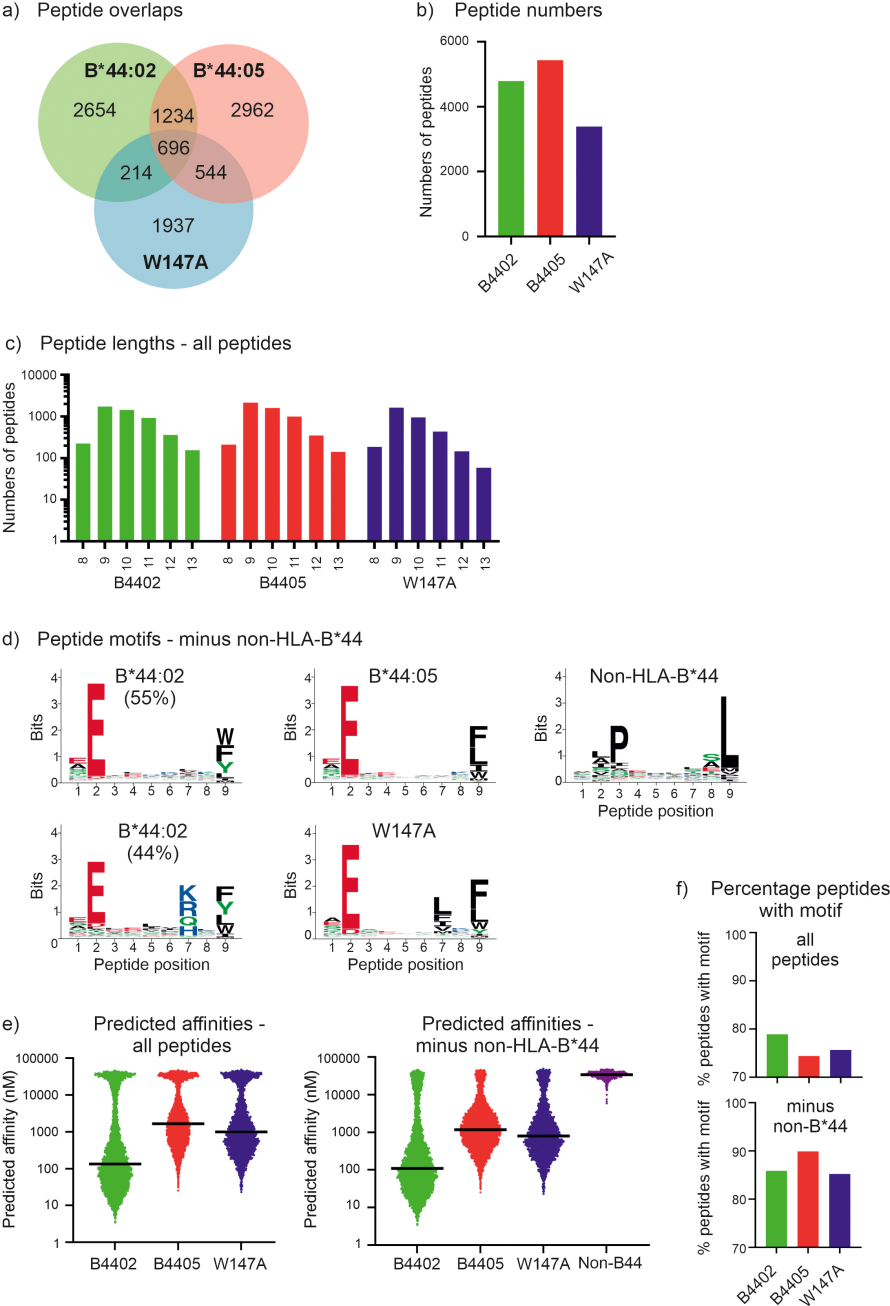
The immunopeptidomes of the tapasin dependent HLA-B*44:02 and HLA-B*44:05-W17A molecules contain greater proportions of high affinity peptides than tapasin independent HLA-B*44:05 molecules. **(a)** Venn diagram showing the number of peptides that were unique to each immunopeptidome, or which were shared. **(b)** Bar graph depicting the number of peptides identified within each immunopeptidome that were 8–13 amino acids long. **(c)** Bar graph depicting the frequency of peptides identified within the immunopeptidomes that were 8–13 amino acids long. **(d)** Motifs representing the immunopeptidomes of the HLA-B*44:02, HLA-B*44:05 and HLA-B*44:05-W17A molecules after Gibbs clustering and omission of potential non-HLA-B*44 binding peptides that did not have glutamic acid at position 2. At each position of a nonameric peptide there is a stack of amino acids with the height of the letter representing the frequency at which that residue was found. Only peptides that were 8–13 amino acids long were considered and were assigned to one (HLA-B*44:05, HLA-B*44:05-W147A, and non-HLA-B*44) or two motifs (HLA-B*44:02, as described previously ref (34)). For HLA-B*44:02, ~6% of peptides had no clear motif, and are not presented. **(e)** Graphs depicting the predicted affinities of the peptides identified within each immunopeptidome with or without omission of the potential non-HLA-B*44 derived peptides. The y axes represent the predicted affinity with nM units, calculated using NetMHCpan as detailed in the methods, with high affinity peptides having low nM values. Each dot represents a peptide, with the median indicated by a black horizontal bar. The left-hand graph shows the predicted affinities when all peptides are included, the right-hand graph shows the predicted affinities when the potential non-HLA-B*44 derived peptides were omitted. **(f)** Graphs depicting the proportion of peptides containing the preferred motif of the indicated MHC-I molecule. This was calculated as the percentage of peptides that contained the preferred motif within each immunopeptidome (“all”, upper plot), or after potential non-HLA-B*44 derived peptides were omitted (“minus non-HLA-B*44”, lower plot). The HLA-B*44:02 and HLA-B*44:05-W147A motifs were glutamic acid at position 2 and any of tryptophan, phenylalanine, tyrosine, leucine, isoleucine or methionine at the C-terminal position. The HLA-B*44:05 motif was glutamic acid at position 2 and any of tryptophan, phenylalanine, leucine, isoleucine or methionine at the C-terminal position.

Most of the peptides identified from each cell line were unique to each immunopeptidome, with only 696 peptides (around 6.8% of all peptides) identified in all three data sets (**Figure 1a**). There were between ~3400-~5500 peptides identified in each immunopeptidome (**Figure 1b**). All three MHC-I allotypes had similar ligand length distributions, with peptides of nine or ten residues being most prevalent (**Figure 1c**). Whilst most peptides showed the P2 glutamic acid anchor residue anticipated for HLA-B*44 ligands, Gibbs cluster analysis of the pooled immunopeptidomes revealed 1228 peptides with a highly distinct motif characterized by enrichment of hydrophobic residues at P2, proline at P3 and leucine at the C-terminus (**Figure 1d**, non-B44). This motif is similar to that reported for HLA-C*01:02 (52) and 66% of these peptides were predicted to bind HLA-C*01:02 by NetMHCpan4.1 (35) (**Supplementary Table 1**). These peptides are likely to be derived from residual HLA-Cw1 expression reported for the 721.220 line (16) which high resolution typing of the related 721.221 cell line reveals to be HLA-C*01:02 (53). Although it should be noted that the majority of these peptides are also predicted to bind non-classical HLA-E (and HLA-G) (**Supplementary Table 1**). Indeed, this cluster contained, VMAPRTLIL which is a well-recognized HLA-E ligand from certain classical HLA leader sequences, including HLA-C*01:02 (54, 55). Thus, except for 51 peptides in this cluster that possessed glutamic acid at P2, we termed these peptides likely non-HLA-B*44 (**Supplementary Table 1**) due to potential contribution by endogenous HLA-C and non-classical HLA of the parental 721.220 cell line.

When the potential non-HLA-B*44 peptides were omitted, we found that, as previously reported (34), the HLA-B*44:02 immunopeptidome was best represented by two peptide motifs, with ~55% of peptides having a motif with almost exclusively glutamic acid at position 2, while tryptophan, phenylalanine, tyrosine, or leucine dominate the C-terminal position (**Figure 1d**, **Supplementary Figure 3** shows the motifs of the molecules without the omission of the potential non-HLA-B*44 peptides). In comparison ~44% of HLA-B*44:02 bound peptides had a motif with similar preferences at positions 2 and 9 but had an additional specificity for amino acids with basic or polar side chains at position 7.

The HLA-B*44:05 immunopeptidome was best represented by a single motif, which was like the most prevalent HLA-B*44:02 motif, with glutamic acid strongly preferred at position 2, and amino acids with hydrophobic side chains preferred at the C-terminal position. The HLA-B*44:05 W147A immunopeptidome closely resembled that of HLA-B*44:05, having a single motif with very similar specificities at positions 2 and 9. One slight difference was a preference for hydrophobic side chains at position 7, particularly leucine, phenylalanine, isoleucine, and valine, which would, presumably, be prevented from binding to wildtype HLA-B*44:05 by the bulky tryptophan side chain of position 147. This analysis therefore confirms that the specificity of the HLA-B*44:05-W147A peptide binding groove is highly similar to that of wild-type HLA-B*44:05.

We next compared the predicted affinities of the peptides recovered from these MHC-I molecules. As we could not definitively determine whether the potential non-HLA-B*44 derived peptides were eluted from HLA-C/E/G molecules co-immunoprecipitated with HLA-B*44 molecules, or if these peptides were low affinity peptides eluted from the HLA-B*44 molecules we compared the effect of omitting these potential non-HLA-B*44 derived peptides from our analysis. We found that regardless of whether the potential non-HLA-B*44 derived peptides were included (**Figure 1e**, left) or omitted (**Figure 1e**, right), the predicted affinity of the median peptide was highest for the peptides identified from HLA-B*44:02 expressing cells and lowest for the HLA-B*44:05 immunopeptidome, with HLA-B*44:05-W147A peptides being intermediate (**Figure 1e** where high affinity peptides have lower nM values than low affinity peptides, **Table 1**). As expected, the peptides likely to derive from non-HLA-B*44 molecules endogenously expressed in the 721.220 cells were predicted to bind HLA-B*44 with very low affinity (**Figure 1e**, non-HLA-B*44, where low affinity peptides have higher nM values than high affinity peptides), and their removal resulted in an increase in the proportion of peptides with the expected B44 anchor residues (**Figure 1f**).

**Table 1:** Descriptive statistics for HLA-B*44:02, HLA-B*44:05 and HLA-B*44:05-W147A immunopeptidomes.

	All peptides			Omitting potential non-HLA-B*44 peptides		
	B*44:02	B*44:05	B*44:05-W147A	B*44:02	*44:05	B*44:05-W147A
Number of values	4798	5436	3391	4410	4499	3011
Median	134	1652	992	109.9	1170	798.7
Std. Deviation	11372	13289	12645	7868	7577	9118
Std. Error of Mean	164.2	180.2	217.1	118.5	113.0	166.2

The affinity of all peptides identified in each immunopeptidomes was predicted (“All peptides”), or after peptides potentially derived from endogenous non-HLA-B*44 molecules were omitted (“Omitting potential non-HLA-B*44 peptides”).

Taken together, these results are consistent with tapasin preferentially skewing the repertoires of peptides presented by tapasin-dependent HLA-B*44:02 and HLA-B*44:05-W147A molecules in favor of the most highly stable peptides, with an increased prevalence of preferred amino acid side chains at positions 2 and 9. This suggests tapasin-dependent MHC-I molecules have less diverse peptide repertoires than tapasin independent MHC-I molecules.

### 3.2 Enhanced tapasin mediated focusing of peptides competing for binding to HLA-B*44:05-W147A compared with HLA-B*44:05

**Figure 2:**
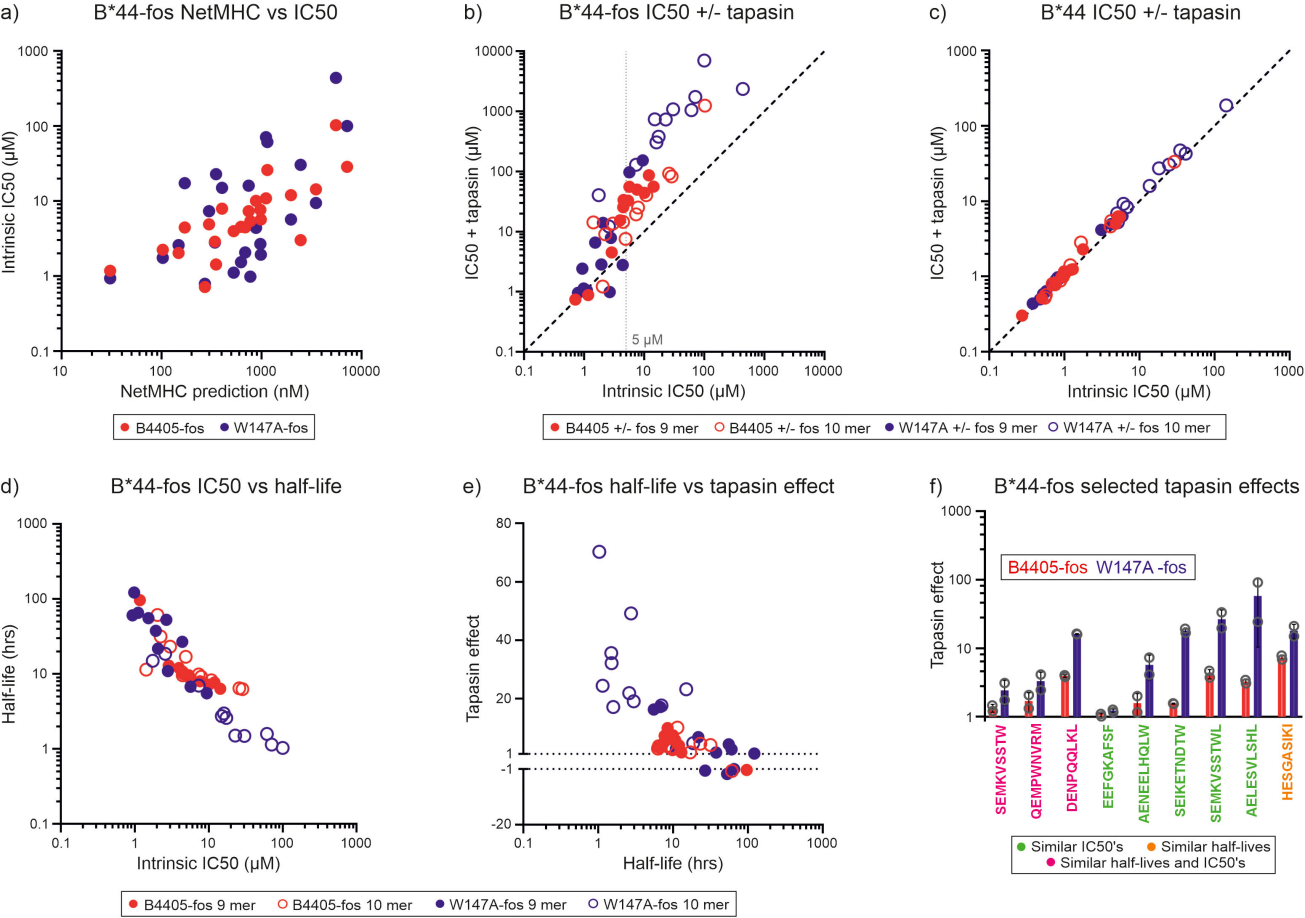
Tapasin-jun-ERp57 focuses the repertoire of peptides selected by HLA-B*44:05-fos and HLA-B*44:05-W147A-fos. **(a)** For HLA-B*44:05-fos and HLA-B*44:05-W147A-fos molecules, the affinities of the peptides used in the competition experiments were predicted and plotted against the measured mean intrinsic IC50 values. The predicted affinities of the competing peptides are represented in nM units, with high affinity peptides having low nM values. The intrinsic IC50 values are the IC50 values measured in the absence of tapasin-jun-Erp57 and are shown in µM units, with high affinity peptides having low µM values. **(b)** Peptide competition experiments were conducted in which unlabeled peptides individually competed against EEFGK^TAMRA^AFSF peptide for binding to either HLA-B*44:05-fos or HLA-B*44:05-W147A-fos in the presence or absence of tapasin-jun-ERp57. Binding of EEFGK^TAMRA^AFSF peptide was measured by fluorescence polarization and IC50 values calculated for each peptide (IC50 value is shown in µM units, with high affinity peptides having low µM values). Each peptide was tested at least twice, and the mean of the replicate experiments is reported. Peptides whose ability to compete for binding is not affected by tapasin-jun-ERp57 will fall along the diagonal dashed line. A faint dashed vertical line indicates a concentration discussed in the text. **(c)** Peptide competition experiments were conducted with HLA-B*44:05 or HLA-B*44:05-W147A molecules without fos leucine zipper sequences in which peptides competed for binding in the presence or absence of tapasin-jun-ERp57. **(d)** The half-lives of the complexes formed between the unlabeled peptides and HLA-B*44:05-fos or HLA-B*44:05-W147A-fos were indirectly measured and plotted against the mean intrinsic IC50 values. Each peptide was tested at least twice, and the mean half-life of the replicate experiments is reported in hours (high affinity peptide-MHC-I complexes have long half-lives). **(e)** The magnitude that tapasin-jun-ERp57 changed the ability of peptides to compete for binding to the HLA-B*44:05-fos or HLA-B*44:05-W147A-fos molecules was calculated and is reported as the “tapasin editing effect” (shortened to “tapasin effect” on the y axis of the graph). When tapasin-jun-ERp57 made a peptide a weaker competitor, this was calculated as the ratio of IC50 measured in the presence of tapasin divided by the intrinsic IC50 and was plotted as a positive number. When tapasin-jun-ERp57 made a peptide a stronger competitor, this was calculated as the ratio of the intrinsic IC50 divided by the IC50 measured in the presence of tapasin and was plotted as a negative number. For each peptide, the tapasin editing effect was plotted against the mean half-life measured for that peptide-MHC-I-fos complex. **(f)** Bar graph showing the “tapasin editing effect” observed for selected peptides competing for binding to HLA-B*44:05-fos or HLA-B*44:05-W147A-fos. Peptides which either competed for binding to HLA-B*44:05-fos or HLA-B*44:05-W147A-fos with similar intrinsic IC50s (peptide sequence in green), or formed complexes with HLA-B*44:05-fos or HLA-B*44:05-W147A-fos with similar half-lives (peptide sequence in orange), or had both similar intrinsic IC50s and similar half-lives (peptide sequence in pink) were selected and the magnitude by which tapasin-jun-ERp57 changed the ability to compete was compared. The tapasin editing effect (“tapasin effect” on y axis) measured in each experiment is shown as an open symbol, with the height of the bar indicating the mean, and the error bar indicating the standard deviation between replicates. To facilitate comparison of how tapasin changed the ability of different peptides to compete for binding, the data is plotted to show the magnitude by tapasin changed their ability to compete irrespective of whether a peptide became a poorer or stronger competitor.

To test whether HLA-B*44:05-W147A selects a higher affinity peptide cargo than HLA-B*44:05 as a result of its interaction with tapasin, we utilized an approach pioneered by Chen and Bouvier to observe tapasin function *in vitro*, in which MHC-I are placed in close proximity to monomeric tapasin molecules, or tapasin-ERp57 heterodimers, via a jun/fos leucine zipper (5). We conducted *in vitro* peptide competition experiments in which each of 24 peptides (twelve 10 mers and twelve 9 mers) that were predicted to cover a wide range of affinities individually competed against the high affinity tetramethyl rhodamine (TAMRA) labelled index peptide EEFGK^TAMRA^AFSF for binding to either HLA-B*44:05-fos or HLA-B*44:05-W147A-fos. EEFGK^TAMRA^AFSF binding and dissociation experiments are shown in **Supplementary Figure 4**. We found for both HLA-B*44:05 and HLA-B*44:05-W147A the affinities that were predicted were correlated with experimentally determined binding affinities as measured by IC50 values (**Figure 2a**, **Table 2**).

**Table 2:** Correlation analyses of the relationships between predicted affinity and measured IC50.

MHC-I molecule	B*44:05	W147A	B*35:01	B*35:03	A*02:01
R^2^	0.4886	0.4304	0.1280	0.3619	0.7957
P value	0.0001	0.0005	0.0861	0.0019	<0.0001

Pearson correlation analyses were performed for the indicated MHC-I molecules to determine whether the predicted affinities correlated with the measured IC50 values. The coefficient of determination (R^2^) is the fraction of variance that is shared between both variables. The p value represents the result of a test of the null hypothesis that the data were sampled from a population in which there is no correlation between the two variables.

Using this approach, we could directly demonstrate tapasin-mediated peptide focusing as tapasin-jun-ERp57 diminished the ability of experimental peptides with lower affinity than the index peptide to compete for binding to both HLA-B*44:05-fos and HLA-B*44:05-W147A-fos (i.e. there was a higher IC50 in the presence of tapasin-jun-ERp57 in **Figure 2b**). This effect was most apparent for peptides with intrinsic IC50s of 5 µM or greater and was dependent on the leucine zipper to tether MHC-I and tapasin-jun-ERp57, as MHC-I molecules lacking the C-terminal fos sequence did not undergo substantial repertoire editing (**Figure 2c**).

Peptide binding half-lives measured for the peptide-MHC-I-fos complexes were inversely related to the intrinsic IC50 measurements (**Figure 2d**). We quantified the magnitude by which tapasin-jun-ERp57 changed the ability of peptides to compete for binding to HLA-B*44:05-fos or HLA-B*44:05-W147A-fos (the “tapasin editing effect”) and plotted this against the peptide-MHC-I complex half-lives (**Figure 2e**). For HLA-B*44:05-fos and HLA-B*44:05-W147A-fos proteins, the magnitude by which tapasin-jun-ERp57 modulated peptide competition increased as the half-life of the peptide-MHC-I-fos complex decreased, such that the least stable peptide-MHC-I-fos complexes experienced the greatest tapasin-jun-ERp57 function and became poorer competitors: i.e. these peptides would be more susceptible to being edited out of the repertoire (**Figure 2e**).

By comparing only those peptides that bound to HLA-B*44:05-fos and HLA-B*44:05-W147A-fos similarly (**Supplementary Figures 5a, b**) we found that, with the exception of EEFGKAFSF peptide, tapasin-jun-ERp57 had a greater influence on peptides competing for binding to HLA-B*44:05-W147A compared with HLA-B*44:05 (**Figure 2f**). This indicates that some low affinity peptides that are selected for presentation by HLA-B*44:05 are likely to be preferentially edited out of the repertoire by introducing the W147A mutation – as a direct result of higher tapasin dependence and more aggressive peptide filtering.

### 3.3 Tapasin modulates the ability of peptides to compete for binding to HLA-B*35:01-fos and HLA-B*35:03-fos

The HLA-B*44:05 and HLA-B*44:05-W147A allotype pair provides a convenient way of isolating the impact of tapasin on peptide repertoire editing, independent of substantial differences in MHC-I peptide binding specificity. We next compared the impact of tapasin on two naturally occurring, related alleles to seek further evidence that the intensity of tapasin-jun-ERp57 optimization increases with the tapasin dependency of an MHC-I allotype. We undertook the same analysis of HLA-B*35:01-fos and HLA-B*35:03-fos, which differ by a single residue at position 116 (HLA-B*35:01: Ser116, HLA-B*35:03: Phe116), and bind similar, although not identical peptide repertoires (56–58). Although both allotypes can efficiently assemble with peptides independently of tapasin, we and others have observed a modest difference in tapasin dependence of these MHC-I allotypes, with HLA-B*35:01 being slightly more independent than HLA-B*35:03 (**Supplementary Figure 1** and refs (14, 15)).

**Figure 3:**
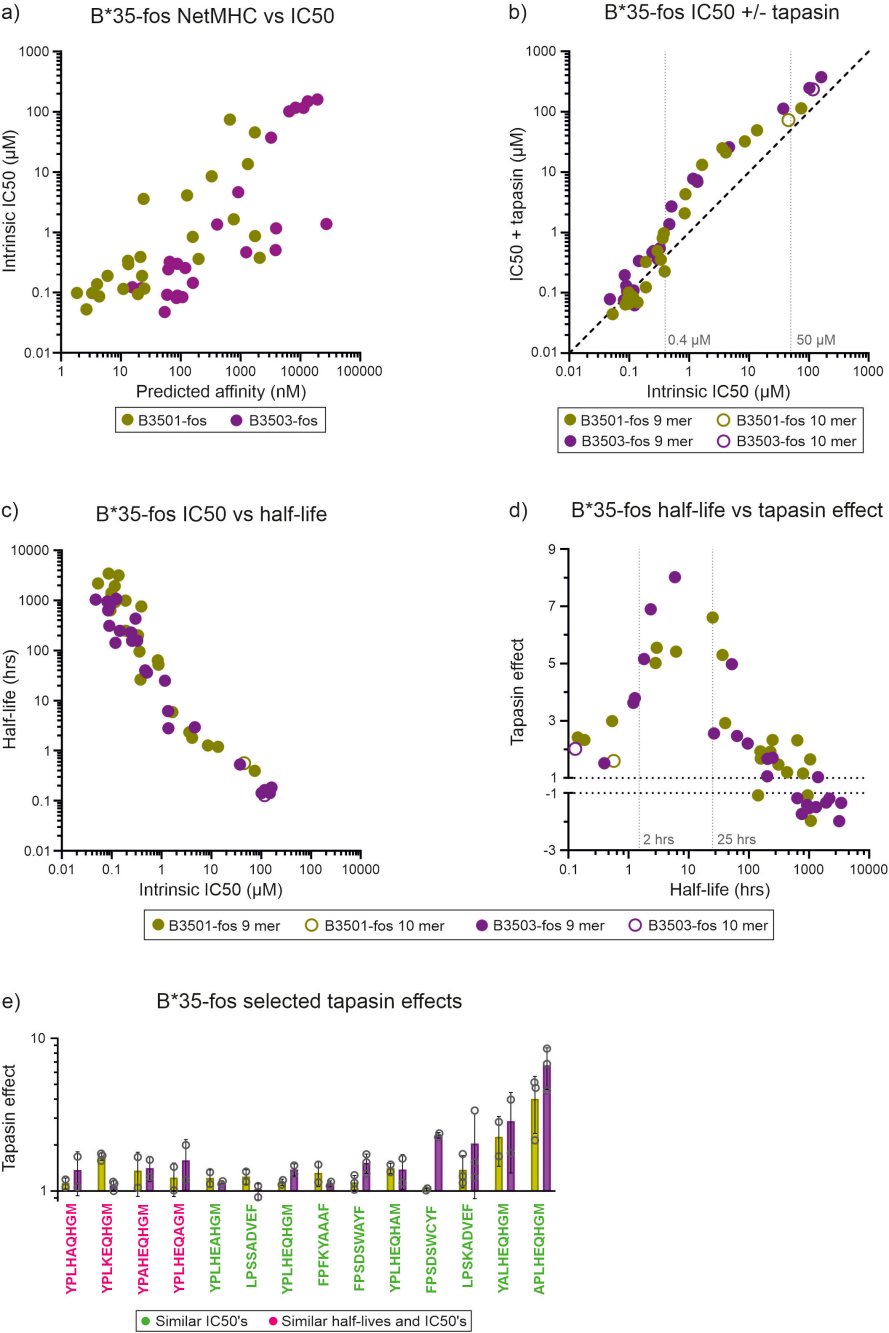
Tapasin-jun-ERp57 focuses the repertoire of peptides selected by HLA-B*35:01-fos and HLA-B*35:03-fos. **(a)** For HLA-B*35:01-fos and HLA-B*35:03-fos molecules, the affinities of the peptides used in the competition experiments were predicted and plotted against the measured mean intrinsic IC50 values as in **Figure 2a**. **(b)** Peptide competition experiments were conducted as described in **Figure 2b** with HLA-B*35:01-fos or HLA-B*35:03-fos molecules. Faint dashed vertical lines indicate concentrations discussed in the text. **(c)** The half-lives of the complexes formed between the unlabeled peptides and HLA-B*35:01-fos or HLA-B*35:03-fos were indirectly measured and plotted against the mean intrinsic IC50 values as in **Figure 2d**. **(d)** The magnitude that tapasin-jun-ERp57 changed the ability of peptides to compete for binding to the HLA-B*35:01-fos or HLA-B*35:03-fos molecules was calculated as before (“tapasin editing effect”) and plotted against the mean half-lives measured for that peptide-MHC-I-fos complex. Faint dashed vertical lines indicate half-lives discussed in the text. **(e)** Bar graph showing the “tapasin editing effect” observed for selected peptides competing for binding to HLA-B*35:01-fos or HLA-B*35:03-fos. Peptides which either competed for binding to HLA-B*35:01-fos or HLA-B*35:03-fos with similar intrinsic IC50s (peptide sequence in green) or had both similar intrinsic IC50s and formed complexes with HLA-B*44:05-fos or HLA-B*44:05-W147A-fos with similar half-lives (peptide sequence in pink) were selected and the magnitude by which tapasin-jun-ERp57 changed the ability to compete was compared and is presented as in **Figure 2f**.

We conducted *in vitro* peptide competition and indirect peptide dissociation experiments using YPLK^TAMRA^EQHGM TAMRA labelled index peptide (YPLK^TAMRA^EQHGM binding and dissociation experiments shown in **Supplementary Figure 6**) and a panel of 24 peptides that covered a wide range of predicted binding affinities for each allotype (**Figure 3a**, **Table 2**). We found that tapasin-jun-ERp57 decreased the ability of intermediate affinity peptides (those with intrinsic IC50 values between 0.4 µM and 50 µM in peptide competition experiments, and half-lives of 2–25 hours in indirect dissociation assays) to compete for binding to both HLA-B*35:01-fos and HLA-B*35:03-fos (**Figures 3b–d**). In contrast, tapasin-jun-ERp57 had comparatively much smaller effects on the competitive abilities of low affinity peptides (intrinsic IC50 values greater than 50 µM and half-lives shorter than 2 hours) which competed poorly against the fluorescent index peptide even in the absence of tapasin, and of high affinity peptides (intrinsic IC50 values smaller than 0.4 µM and half-lives longer than 25 hours), which competed potently against the fluorescent index peptide. Fourteen peptides bound to HLA-B*35:01-fos and HLA-B*35:03-fos with similar affinity (**Supplementary Figures 5c, d**), and we found that tapasin-jun-ERp57 had a greater influence on the ability of many of these 14 peptides to compete for binding to HLA-B*35:03-fos than was apparent for HLA-B*35:01-fos (**Figure 3e**). This was consistent with the slightly greater tapasin dependence of HLA-B*35:03 observed by us and others (**Supplementary Figure 1** and ref (14)) and consistent with HLA-B*44:05-W147A receiving greater optimization from tapasin than HLA-B*44:05.

### 3.4 TAPBPR modulates the ability of peptides to compete for binding to HLA-A*02:01-fos

**Figure 4:**
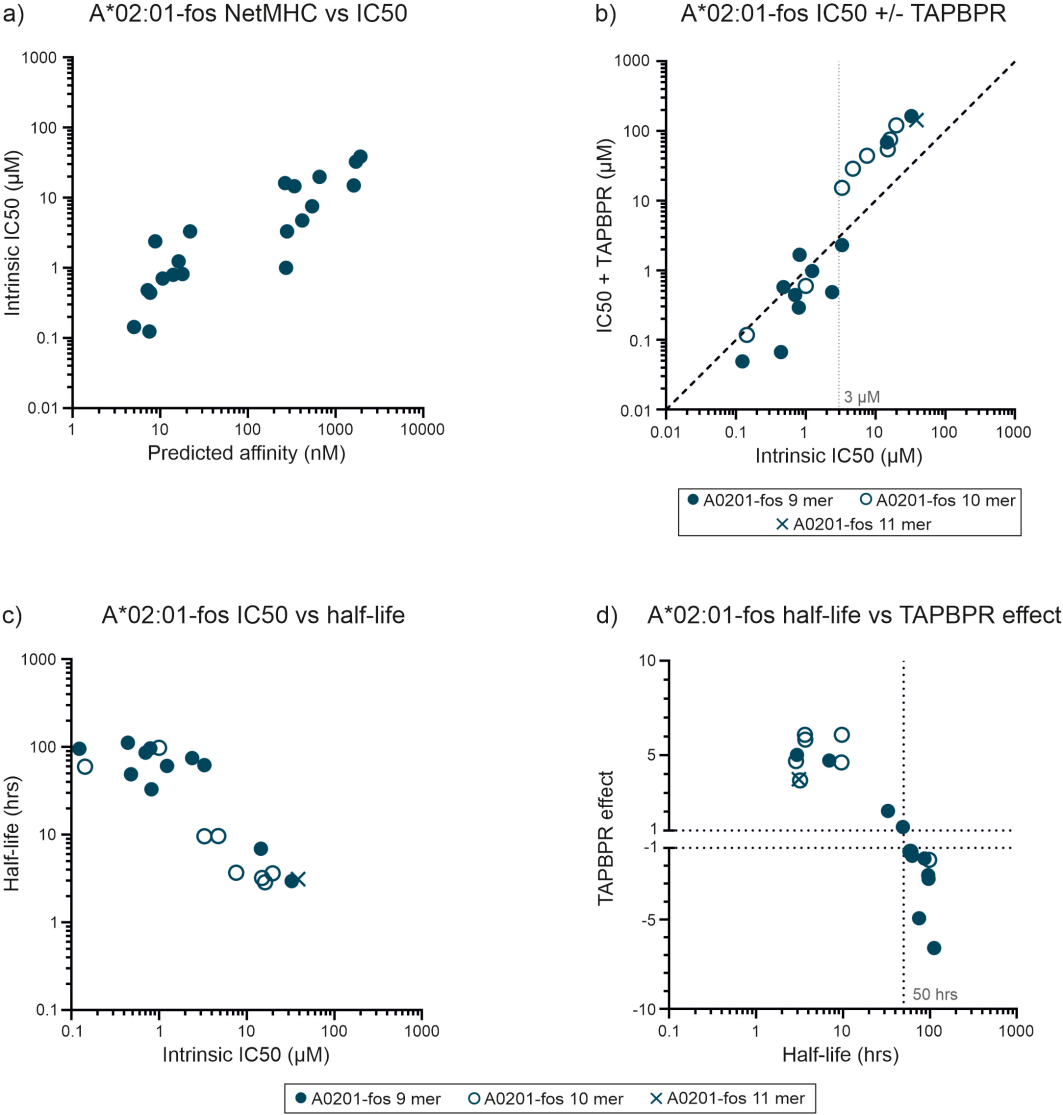
TAPBPR focuses the repertoire of peptides selected by HLA-A*02:01-fos. **(a)** For HLA-A*02:01-fos molecules, the affinities of the peptides used in the competition experiments were predicted and plotted against the measured mean intrinsic IC50 values as in **Figure 2a**. **(b)** Peptide competition experiments were conducted in the presence or absence of TAPBPR. Each peptide was tested at least twice, and the mean of the replicate experiments is reported. A faint dashed vertical line indicates a concentration discussed in the text. **(c)** The half-lives of the complexes formed between the unlabeled peptides and HLA-A*02:01-fos were indirectly measured and plotted against the mean intrinsic IC50 values as in **Figure 2d**. **(d)** The magnitude that TAPBPR changed the ability of peptides to compete for binding to the HLA-A*02:01-fos molecules was calculated (in the same manner as for tapasin) and plotted as the “TAPBPR editing effect” as in **Figure 2e** (shortened to “TAPBPR effect” on the y axis of the graph). This was plotted against the mean half-lives measured for the peptide-MHC-I-fos complexes on the x axis.

We, and others, have previously shown that the tapasin orthologue, TAPBPR, which is not part of the peptide loading complex and most likely acts on peptide-MHC-I complexes released from the PLC as a further quality control checkpoint, also has a peptide editing function similar to tapasin (6–8). We therefore sought to determine whether TAPBPR might focus the peptide repertoire of HLA-A*02:01, a relatively tapasin independent allotype that, unlike HLA-B*44:02, HLA-B*44:05, HLA-B*35:01 and HLA-B*35:03 receives considerable benefit from TAPBPR mediated peptide editing (7, 8, 18). We carried out peptide competition experiments with a panel of 20 peptides that were predicted to cover a wide range of affinities (**Figure 4a**, **Table 2**) and the high affinity FLPSDC^TAMRA^FPSV index peptide (FLPSDC^TAMRA^FPSV binding and dissociation experiments shown in **Supplementary Figure 7**). TAPBPR modulated the ability of peptides to compete for binding to HLA-A*02:01 (**Figures 4b–d**). TAPBPR made low affinity peptides, with intrinsic IC50 values of 3 µM or greater, and half-lives of less than 50 hours, substantially poorer competitors for binding. Conversely, TAPBPR made most peptides with intrinsic IC50 values less than 3 µM, and half-lives of 50 hours or longer, stronger competitors for binding to HLA-A*02:01 (**Figure 4b–d**). The divergent effects that TAPBPR had on the ability of peptides to compete for binding to HLA-A*02:01 suggests that the index FLPSDC^TAMRA^FPSV peptide has an IC50 value of around 3 µM, (approximately 20-fold lower than was measured for unlabeled FLPSDCFPSV [0.14 µM]). Thus, we found that TAPBPR can also modulate the peptide repertoire in an analogous fashion to tapasin.

## 4 Discussion

The highly polymorphic nature of MHC-I molecules has long been known to define peptide binding specificity and, at least in mammals, dependence upon tapasin and TAPBPR for the acquisition of high affinity peptides. Recently, it has been suggested that the tapasin dependence of human MHC-I allotypes is inversely correlated with the diversity of their immunopeptidome (14, 27, 59). Our analysis of the HLA-B*44:02, HLA-B*44:05 and HLA-B*44:05-W147A immunopeptidomes provides direct evidence that tapasin dependent MHC-I allotypes generally bind peptide repertoires that are enriched in higher affinity peptides, closely complimenting the specificities of MHC-I anchor pockets. In comparison, tapasin independent MHC-I allotypes generally bind peptide repertoires that have lower average affinity and are less well suited to MHC-I anchor pocket specificities, and are likely to contain a greater diversity of peptide sequences. This suggests, that when tapasin is allowed to engage HLA-B*44:02 molecules or HLA-B*44:05 molecules in cells because of either aspartic acid at position 116 (HLA-B*44:02) or the W147A mutation (HLA-B*44:05-W147A), this results in more aggressive peptide focusing.

It is thought that MHC-I molecules transition between “closed” peptide bound states with low free energy and “open” peptide receptive states with higher free energy, with iterations of this process underpinning peptide exchange (22, 23, 29, 60–65). The mechanism by which tapasin independent MHC-I allotypes can self-edit their peptide repertoire remains to be determined. One possibility is that MHC-I allotypes like HLA-B*44:05 can independently self-edit their peptide repertoire because they have an intrinsic ability to adopt and transition between open and closed states. Indeed, as differences in MHC-I-fos-tapasin-jun-ERp57 binding affinities are unlikely in the context of an artificially tethered interaction, a potential mechanistic explanation for the greater tapasin optimization experienced by tapasin-dependent (HLA-B*44:05-W147A-fos or HLA-B*35:03-fos) molecules is that for tapasin dependent molecules there is a slower rate of transition from open to closed states, as has been suggested previously (29). Thus, tapasin dependent MHC-I allotypes have less intrinsic potential to transition between states and are consequently hard-wired to experience greater benefit from tapasin when it is available.

An alternate mechanism by which the W147A mutation increased the tapasin dependence of HLA-B*44:05 involves the hydrogen bond formed between the tryptophan at position 147, which is highly conserved in classical MHC-I, and the penultimate carbonyl group of the peptide. It is likely that in the absence of this hydrogen bond, there is increased conformational flexibility surrounding the C-terminal portion of the peptide and in the α2–1 sub-helix, which are key interaction surfaces for tapasin (1, 66–68) or TAPBPR (69, 70). The abrogation of this hydrogen bond tethering peptide to the MHC-I peptide binding groove is likely to have facilitated a higher affinity interaction with tapasin, allowing greater potential for tapasin assisted peptide editing to occur (65). A third possibility is that the ability to self-edit peptide repertoires involves the displacement of low affinity peptides from the peptide binding groove without substantial rearrangements of protein domains. In such a scenario, our experiments indicate that the combination of tryptophan at position 147 and tyrosine at position 116 are integral to the self-editing ability of HLA-B*44:05 while the combination of tryptophan and aspartic acid at these positions does not permit self-editing for HLA-B*44:02. However, the mechanism by which these residues are involved in self-editing remains to be determined. While molecular models of MHC-I self-editing have been proposed based on analysis of selected MHC-I allotypes (29, 71–76), these models do not provide a universal mechanism of self-editing that is applicable for all tapasin independent MHC-I allotypes, and it is possible that different mechanisms may operate in different MHC-I allotypes.

There may be multiple physiologically relevant consequences of variable degrees of repertoire editing by tapasin and TAPBPR. For tapasin dependent MHC-I allotypes, and for those MHC-I molecules that bind TAPBPR strongly, the extent of peptide focusing is likely to change in line with the expression levels of tapasin and TAPBPR, along with other components of the MHC-I antigen processing and presentation pathway. While cytokines released in response to inflammation may enhance MHC-I mediated antigen presentation, viral immune evasion proteins may target key proteins, including tapasin, to avoid MHC-I antigen presentation (77, 78). Similarly, some cancers lose expression of proteins involved in MHC-I mediated antigen presentation, including tapasin, generally leading to tumor progression and poorer prognoses (20, 79, 80). Thus, those MHC-I molecules that can independently optimize their peptide repertoire may be less susceptible to down regulation of tapasin.

Our data supports recent suggestions that in humans, tapasin dependence and immunopeptidome diversity are inversely correlated (14, 27, 59). Thus, tapasin dependency is likely to result in a more focused profile of presented peptides in which fewer, higher affinity peptides are presented at relatively higher abundance. There are also substantial differences in the diversity of MHC-I immunopeptidomes presented by different chicken MHC-I allotypes, with MHC-I immunopeptidome diversity being correlated, not with tapasin dependence, but with the structure of the MHC-I peptide binding groove and the specificity of the co-evolving polymorphic TAP peptide transporters (27, 59, 81, 82). Importantly, in both chickens and humans, diverse MHC-I immunopeptidomes have been shown to correlate with resistance to certain infectious pathogens (14, 27, 59). Additionally, vaccination studies in rhesus macaques have shown that presentation of a diverse pool of peptides by non-classical Mamu-E MHC-I molecules, equivalent to HLA-E in humans, resulted in enhanced immune responses (83). Thus, the protective benefit of diverse MHC-I immunopeptidomes appears to be an evolutionarily conserved feature of MHC-I molecules. MHC-I molecules with promiscuous peptide binding specificities, or the ability to select and present a broad peptide repertoire in the absence of tapasin/TAPBPR might be considered survival “generalists” (59). By comparison, MHC-I molecules with fastidious peptide binding specificities, or high dependency on tapasin/TAPBPR for repertoire editing may be beneficial for responding to new, and perhaps especially virulent pathogens, and therefore may be classified as survival “specialists” (59). Similarly, in the context of tumor immunology, a generalist approach (e.g. low tapasin dependence, mild editing) may be more protective against tumors that express multiple tumor specific neoepitopes, whereas for those that express a paucity of neoepitopes a more specialist approach (e.g. high tapasin dependence, aggressive editing) may be preferable.

In conclusion, our data are consistent with a scenario in which the dependence of individual MHC-I allotypes upon tapasin for optimal peptide selection underpins the diversity of their immunopeptidomes (14, 27, 59). Our *in vitro* observations of tapasin-jun-ERp57 and TAPBPR mediated peptide focusing illustrate how tapasin or TAPBPR filters the immunopeptidome according to individual peptide-MHC-I complex stability.

## Data Availability

The datasets presented in this study can be found in online repositories. The names of the repository/repositories and accession number(s) can be found below: https://www.ebi.ac.uk/pride/archive/, PXD054743 10.6019/PXD054743.

## Supplementary material

Supplementary Methods 1 DNA constructs encoding HLA-B*35:01 or HLA-B35:03* in the pMSCVneo plasmid were kindly provided by Prof M. Raghavan (15) and sub-cloned into the pMCFRpuro plasmid. Stable transfectants of tapasin-deficient 721.220 cells, or tapasin-reconstituted 721.220-tapasin cells, expressing HLA-B*35:01, HLA-B*35:03 were generated as described (29). **MHC-I pulse chase thermostability assays.** MHC-I thermostability assays were performed essentially as described (2). In brief, tapasin-deficient.220 cells, or tapasin-reconstituted.220-tapasin cells, expressing the indicated MHC-I molecules were radiolabeled with 10 μ Ci/ml ^35^S Translabel for six minutes, chased for the indicated period, before the cells were lysed. The lysates were heated to 37°C for 12 minutes, before being cooled, and MHC-I-β_2_m complexes immunoprecipitated with W6/32 antibody. The eluted proteins were separated by SDS PAGE, and the gels were stained with Coomassie brilliant blue and an image of the gel taken, before the gels were dried and exposed to phosphor screens. The intensities of the radiolabeled heavy chain bands were measured using Personal Molecular Imager FX and quantified using Quantity One software and normalized according to the corresponding intensity of immunoprecipitated W6/32 antibody heavy chain bands in the Coomassie stained gel image. **Flow cytometry measurements of MHC-I cell surface expression.** Cells were stained with W6/32 antibody followed by anti-mouse FITC secondary antibody in the dark, washed and analyzed on a FACSCalibur flow cytometer (BD Biosciences). **MHC-I pulse chase maturation assays.** MHC-I pulse chase maturation assays were performed as described (2, 29). **MHC-I peptide stabilization assays.** Tapasin-deficient.220 cells expressing the indicated MHC-I molecules were incubated at 37°C for 40 minutes in 4 ml of methionine and cysteine free RPMI media that was supplemented with 10% dialyzed FCS and 2 mM L-glutathione. The cells were radiolabeled with 7.8 MBq of ^35^S Translabel for 30 minutes at 37°C before the radiolabeling was quenched by the addition of 20 ml ice cold PBS, and the cells lysed. The lysates were divided into five aliquots and incubated for 30 minutes on ice with either 0, 1, 2, 5 or 20 µM of the following known HLA-B*44 binding peptides (FEDLRVLSF, VETPIRNEW, TENGSFVAGY, EENLLDFVRF or SEIDTVAKY, refs (84–87)). MHC-I molecules were immunoprecipitated using W6/32 antibody as detailed above and separated by SDS PAGE gels.

Supplementary Figure 1 The HLA-B*35:01, HLA-B*35:03, HLA-B*44:02, HLA-B*44:05 and HLA-B*44:05-W147A MHC-I molecules differ in their dependence upon tapasin. **(a)** Representative images of ^35^S pulse labelled MHC-I thermostability assays of the indicated MHC-I molecules expressed in tapasin deficient 721.220 cells (- tapasin) or tapasin reconstituted 721.220-tapasin cells (+ tapasin). Only the portion of the gel showing the heavy chain band is shown. **(b)** Bar graph depicting how much tapasin improved the thermostability of the indicated MHC-I molecules. This was calculated as the ratio of the percentage of thermostable MHC-I recovered in the presence of tapasin divided by the percentage of thermostable MHC-I recovered in the absence of tapasin after a chase of 120 minutes. Dots represent results from individual experiments, and vertical lines depict the standard deviation observed between experiments. The data for HLA-B*44:02, HLA-B*44:05 and HLA-B*44:05-W147A is published in ref (29). **(c)** Bar graph showing the cell surface expression level of the indicated MHC-I molecules transfected into tapasin deficient 721.220 cells (- tapasin) or tapasin reconstituted 721.220-tapasin cells (+ tapasin) following staining with W6/32 antibody and flow cytometry analysis. Dots represent the mean fluorescent intensity measured in an experiment, and vertical lines depict the standard deviation observed between experiments. The data for HLA-B*44:02, HLA-B*44:05 and HLA-B*44:05-W147A is published in ref (29). **(d)** Representative images of ^35^S pulse chase MHC-I maturation assays of the indicated MHC-I molecules expressed in tapasin deficient 721.220 cells (- tapasin) or tapasin reconstituted 721.220-tapasin cells (+ tapasin). W6/32 immunoprecipitated samples were digested (+) or mock digested (-) with endoglycosidase H (endo H). ER and ES denote immunoprecipitated MHC-I molecules that are resistant (ER) or sensitive (ES) to digestion with endoglycosidase H. **(e)** Graphs depicting the percentage of immunoprecipitated MHC-I molecules that were resistant to digestion with endoglycosidase H at each chase point in the absence (left) or presence of tapasin (right). Vertical error bars denote the standard deviation observed between experiments. The maturation of HLA-B*44:02, HLA-B*44:05 and HLA-B*44:05-W147A in 721.220 or 721.220-tapasin cells is published in ref (29).

Supplementary Figure 2 Peptide stabilization assays with tapasin-deficient cells expressing HLA-B*44:02, HLA-B*44:05 or HLA-B*44:05-W147A. MHC-I peptide stabilization assays of the indicated MHC-I molecules expressed in tapasin deficient 721.220 cells. The cells were radiolabeled before the cells were lysed. Aliquots of lysates were incubated with the indicated peptide for 30 minutes before MHC-I molecules were immunoprecipitated and separated by SDS PAGE. Only the portion of the gel showing the heavy chain band is shown. Graphs show the density of the heavy chain bands for the indicated MHC-I molecules. The anomalous data point for HLA-B*44:05-W147A incubated with 2 µM FEDLRVLSF (denoted with an asterisk above) was omitted from the graph for clarity.

Supplementary Figure 3 Analysis of the immunopeptidomes without peptides potentially derived from non-HLA-B*44 molecules. Motifs representing the immunopeptidomes of the HLA-B*44:02, HLA-B*44:05, HLA-B*44:05-W17A molecules without any peptides being omitted from the analysis. Peptides were assigned to one (HLA-B*44:05 and HLA-B*44:05-W147A) or two motifs (HLA-B*44:02, as described previously ref (34)). For HLA-B*44:02, ~11% of peptides had no clear motif, and are not presented.

Supplementary Figure 4 Peptide binding and peptide dissociation experiments with HLA-B*44:05-fos and HLA-B*44:05-W147A-fos in the presence or absence of tapasin-jun-ERp57. **(a)** Peptide binding experiment in which 160 nM HLA-B*44:05-fos was supplemented with 3.2 µM β_2_-microglobulin and UV exposed, before being added to 2 nM EEFGK*AFSF peptide in the presence or absence of 300 nM tapasin-jun-ERp57. Fluorescence polarization was measured at 25°C. **(b)** Peptide binding experiment with HLA-B*44:05-W147A-fos as in **Supplementary Figure 4a**. **(c)** Peptide dissociation experiment in which 160 nM HLA-B*44:05-fos was supplemented with 3.2 µM β_2_-microglobulin, UV exposed and incubated overnight with 2 nM EEFGK*AFSF peptide. The next day, 66.7 µM EENLLDFVRF peptide competitor was added in the presence or absence of 300 nM tapasin-jun-ERp57. Fluorescence polarization was measured at 25°C. **(d)** Peptide dissociation experiment with HLA-B*44:05-W147A-fos as in **Supplementary Figure 4c**.

Supplementary Figure 5 Identification of similar binding peptides. **(a)** The mean intrinsic IC50 value of each peptide competing for binding to HLA-B*44:05-fos is shown on the x-axis, while the mean intrinsic IC50 value measured for each peptide competing for binding to HLA-B*44:05-W147A-fos is shown on the y-axis. To identify peptides with similar abilities to compete for binding to both HLA-B*44:05-fos and HLA-B*44:05-W147A-fos, the ratio by which the intrinsic IC50s differed was calculated. Peptides with an intrinsic IC50 ratio of less than 2 are shown in green or shown in pink if the peptides also have a half-life ratio of less than 2, all other peptides are shown in grey. **(b)** The mean half-lives of the peptide-HLA-B*44:05-fos complexes are shown on the x-axis, while the mean half-lives of the peptide-HLA-B*44:05-W147A-fos complexes are shown on the y-axis. To identify peptides that formed complexes with HLA-B*44:05-fos and HLA-B*44:05-W147A-fos with similar half-lives, the ratio by which the half-lives differed was calculated. Peptides with a half-life ratio of less than 2 are shown in orange or shown in pink if the peptides also have an intrinsic IC50 ratio of less than 2, all other peptides are shown in grey. **(c)** The mean intrinsic IC50 value of each peptide competing for binding to HLA-B*35:01-fos is shown on the x-axis, while the mean intrinsic IC50 value measured for each peptide competing for binding to HLA-B*35:03-fos is shown on the y-axis. Peptides with similar intrinsic IC50 ratios were calculated as in **Supplementary Figure 5a**. **(d)** The mean half-lives of the peptide-HLA-B*35:01-fos complexes are shown on the x-axis, while the mean half-lives of the peptide-HLA-B*35:03-fos complexes are shown on the y-axis. Peptides with similar half-life ratios were calculated as in **Supplementary Figure 5b**.

Supplementary Figure 6 Peptide binding and peptide dissociation experiments with HLA-B*35:01-fos and HLA-B*35:03-fos in the presence or absence of tapasin-jun-ERp57. **(a)** Peptide binding experiment in which 225 nM HLA-B*35:01-fos was supplemented with 4.5 µM β_2_-microglobulin and UV exposed, before being added to 3 nM YPLK*EQHGM peptide in the presence or absence of 300 nM tapasin-jun-ERp57. Fluorescence polarization was measured at 25°C. **(b)** Peptide binding experiment with HLA-B*35:03-fos as in **Supplementary Figure 6a**. **(c)** Peptide dissociation experiment in which 225 nM HLA-B*35:01-fos was supplemented with 4.5 µM β_2_- microglobulin and UV exposed and incubated overnight with 3 nM YPLK*EQHGM peptide. The next day, 66.7 µM YPLHEQHGM peptide competitor was added in the presence or absence of 300 nM tapasin-jun-ERp57. Fluorescence polarization was measured at 25°C. **(d)** Peptide dissociation experiment with HLA-B*35:03-fos as in **Supplementary Figure 6c**.

Supplementary Figure 7 Peptide binding and peptide dissociation experiments with HLA-A*02:01-fos in the presence or absence of TAPBPR. **(a)** Peptide binding experiment in which 50 nM HLA-A*02:01-fos was supplemented with 1 µM β_2_-microglobulin and UV exposed, before being added to 2 nM FLPSDC*FPSV peptide in the presence or absence of 300 nM TAPBPR. Fluorescence polarization was measured at 25°C. **(b)** Peptide dissociation experiment in which 50 nM HLA-A*02:01-fos was supplemented with 1 µM β_2_-microglobulin and UV exposed and incubated overnight with 2 nM FLPSDC*FPSV peptide. The next day, 66.7 µM FLPSDCFPSV peptide competitor was added in the presence or absence of 300 nM TAPBPR and fluorescence polarization was measured at 25°C.

Supplementary Table 1 Peptide lists and analysis of potential non-HLA-B44 peptides. The first three tabs of the spreadsheet provide lists of peptides that are 8–13 amino acids and non-redundant by sequence (i.e. modifications were not considered) that were identified within each immunopeptidome (B4402 peptides, B4405 peptides, W147A peptides). The potential non-HLA-B*44 peptides identified by Gibbs clustering are designated 1 for potential non-HLA-B*44 ligands or 0 for potential HLA-B*44 ligands. The fourth tab provides a list of 1228 potential non-HLA-B*44 binding peptides that were identified by pooling and clustering all peptides (NonB44 cluster analysis). This tab provides an analysis of the potential sources of these peptides and their predicted binding affinity to HLA-B*44, HLA-C*01:02, HLA-E or HLA-G molecules. The fifth tab (Non B44 peptides) provides a list of 1177 potential non-HLA-B*44 binding peptides, i.e. peptides identified by clustering that do not have glutamic acid at position 2. The sixth tab (Overlaps) details the extent to which peptides are shared between immunopeptidomes or were unique. The analysis was conducted including potential non-B*44 peptides and is replicated omitting these peptides.
