# Supplementary figures and images for: Evidence of focusing the MHC class I immunopeptidome by tapasin
Source: Front Immunol. 2025 May 8;16:1563789. doi: 10.3389/fimmu.2025.1563789 (PMC12094946; doi:10.3389/fimmu.2025.1563789)

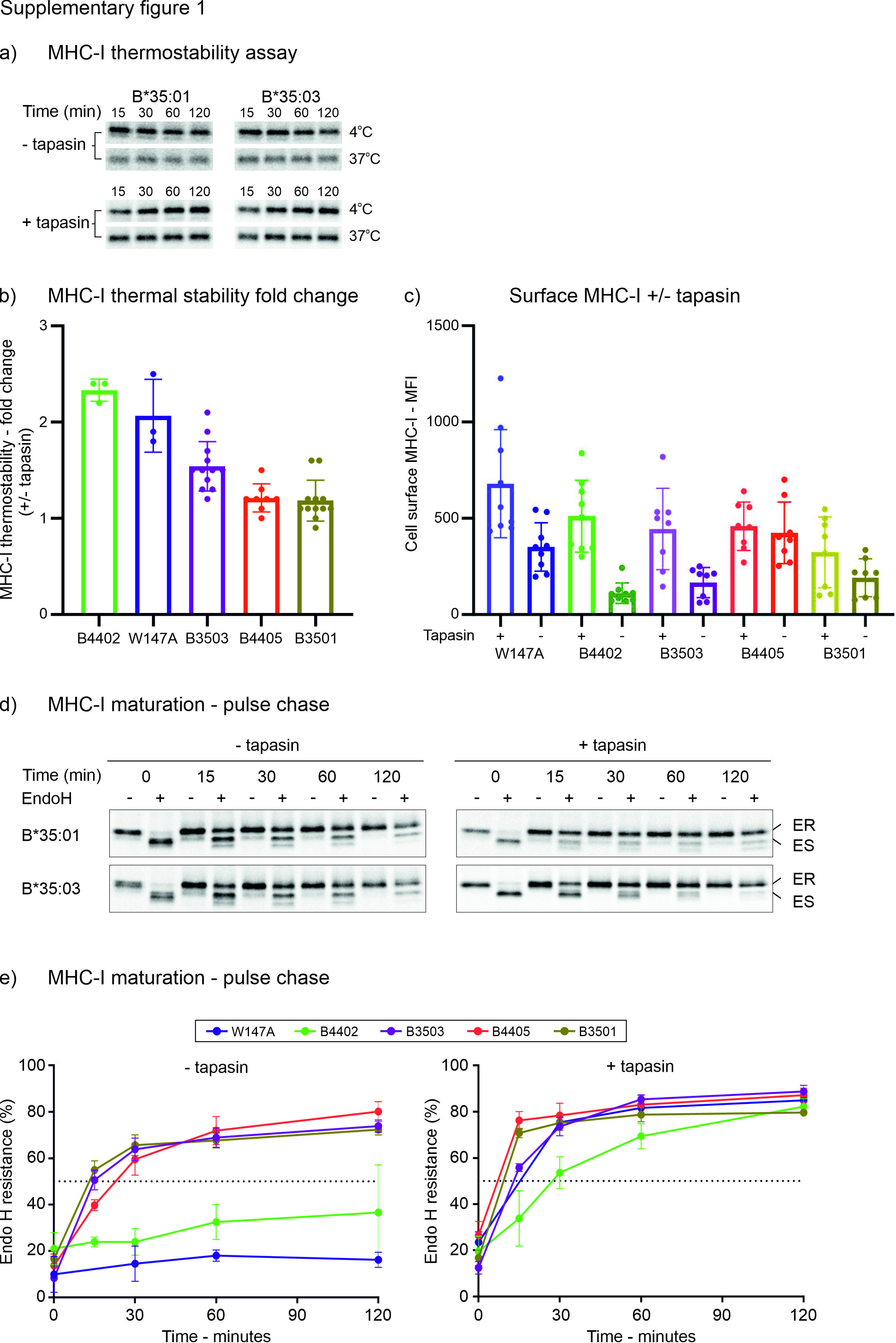

Supplement: Supplementary Methods 1 — DNA constructs encoding HLA-B35:01 or HLA-B35:03 in the pMSCVneo plasmid were kindly provided by Prof M. Raghavan (15) and sub-cloned into the pMCFRpuro plasmid. Stable transfectants of tapasin-deficient 721.220 cells, or tapasin-reconstituted 721.220-tapasin cells, expressing HLA-B*35:01, HLA-B*35:03 were generated as described (29). MHC-I pulse chase thermostability assays. MHC-I thermostability assays were performed essentially as described (2). In brief, tapasin-deficient.220 cells, or tapasin-reconstituted.220-tapasin cells, expressing the indicated MHC-I molecules were radiolabeled with 10 μ Ci/ml 35S Translabel for six minutes, chased for the indicated period, before the cells were lysed. The lysates were heated to 37°C for 12 minutes, before being cooled, and MHC-I-β2m complexes immunoprecipitated with W6/32 antibody. The eluted proteins were separated by SDS PAGE, and the gels were stained with Coomassie brilliant blue and an image of the gel taken, before the gels were dried and exposed to phosphor screens. The intensities of the radiolabeled heavy chain bands were measured using Personal Molecular Imager FX and quantified using Quantity One software and normalized according to the corresponding intensity of immunoprecipitated W6/32 antibody heavy chain bands in the Coomassie stained gel image. Flow cytometry measurements of MHC-I cell surface expression. Cells were stained with W6/32 antibody followed by anti-mouse FITC secondary antibody in the dark, washed and analyzed on a FACSCalibur flow cytometer (BD Biosciences). MHC-I pulse chase maturation assays. MHC-I pulse chase maturation assays were performed as described (2, 29). MHC-I peptide stabilization assays. Tapasin-deficient.220 cells expressing the indicated MHC-I molecules were incubated at 37°C for 40 minutes in 4 ml of methionine and cysteine free RPMI media that was supplemented with 10% dialyzed FCS and 2 mM L-glutathione. The cells were radiolabeled with 7.8 MBq of 35S Translabel for 30 minut [file Image1.jpeg]

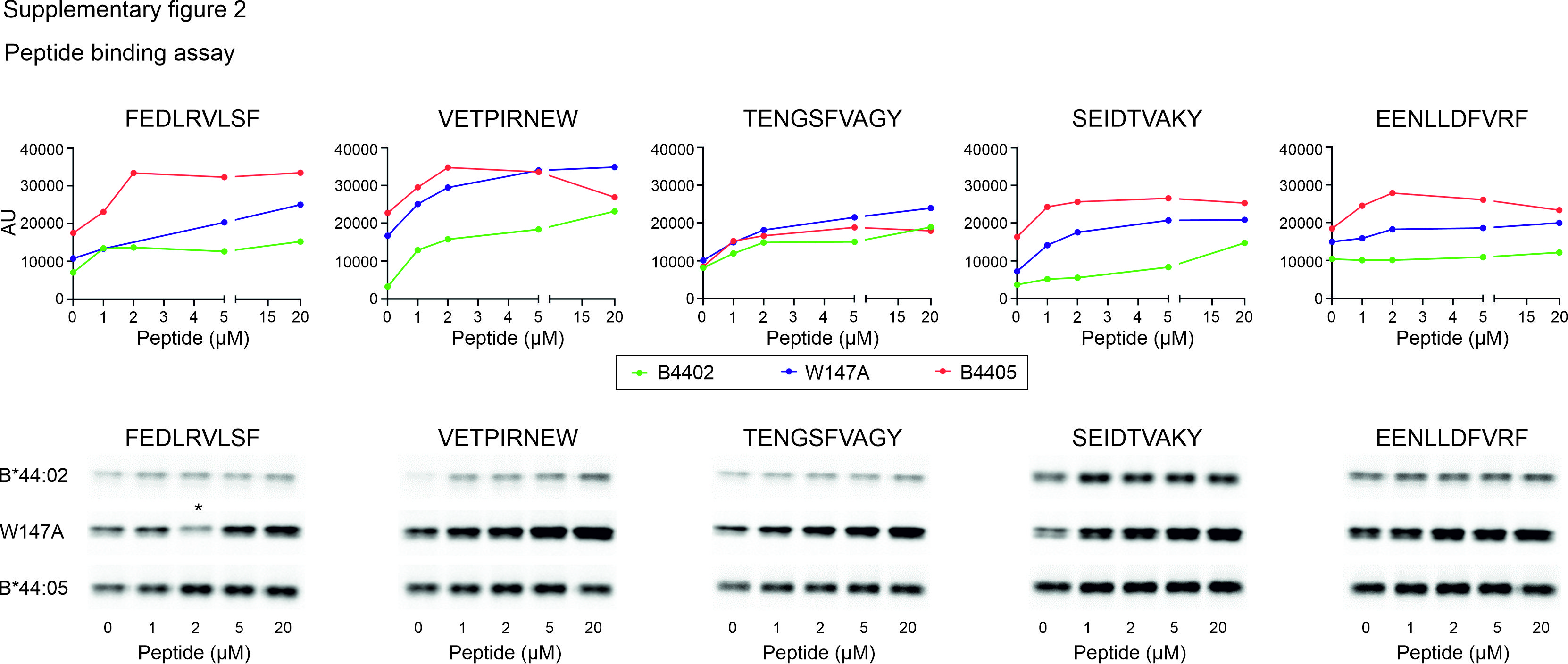

Supplement: Supplementary Figure 2 — Peptide stabilization assays with tapasin-deficient cells expressing HLA-B*44:02, HLA-B*44:05 or HLA-B*44:05-W147A. MHC-I peptide stabilization assays of the indicated MHC-I molecules expressed in tapasin deficient 721.220 cells. The cells were radiolabeled before the cells were lysed. Aliquots of lysates were incubated with the indicated peptide for 30 minutes before MHC-I molecules were immunoprecipitated and separated by SDS PAGE. Only the portion of the gel showing the heavy chain band is shown. Graphs show the density of the heavy chain bands for the indicated MHC-I molecules. The anomalous data point for HLA-B*44:05-W147A incubated with 2 µM FEDLRVLSF (denoted with an asterisk above) was omitted from the graph for clarity. [file Image2.jpeg]

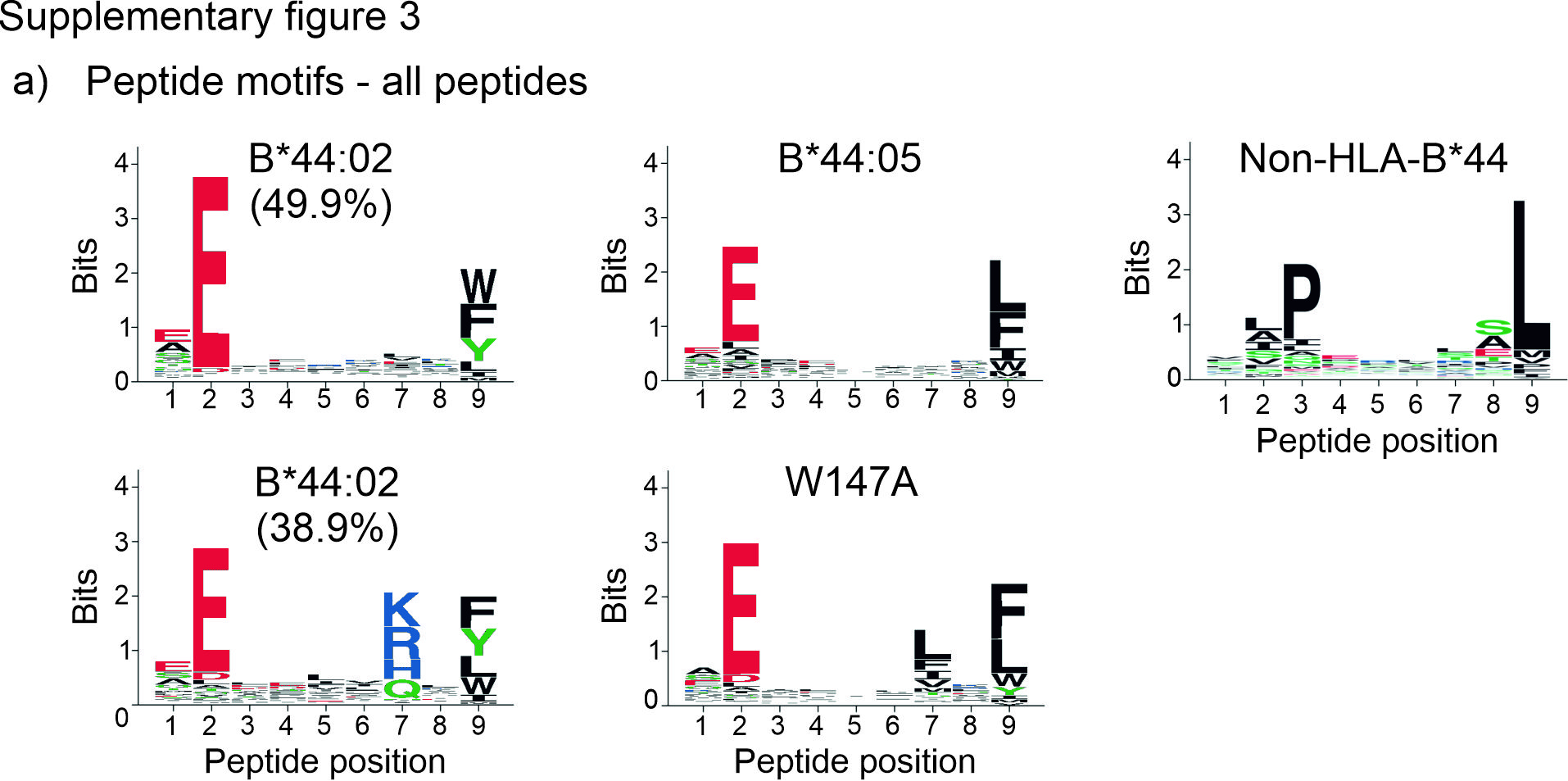

Supplement: Supplementary Figure 3 — Analysis of the immunopeptidomes without peptides potentially derived from non-HLA-B*44 molecules. Motifs representing the immunopeptidomes of the HLA-B*44:02, HLA-B*44:05, HLA-B*44:05-W17A molecules without any peptides being omitted from the analysis. Peptides were assigned to one (HLA-B*44:05 and HLA-B*44:05-W147A) or two motifs (HLA-B*44:02, as described previously ref (34)). For HLA-B*44:02, ~11% of peptides had no clear motif, and are not presented. [file Image3.jpeg]

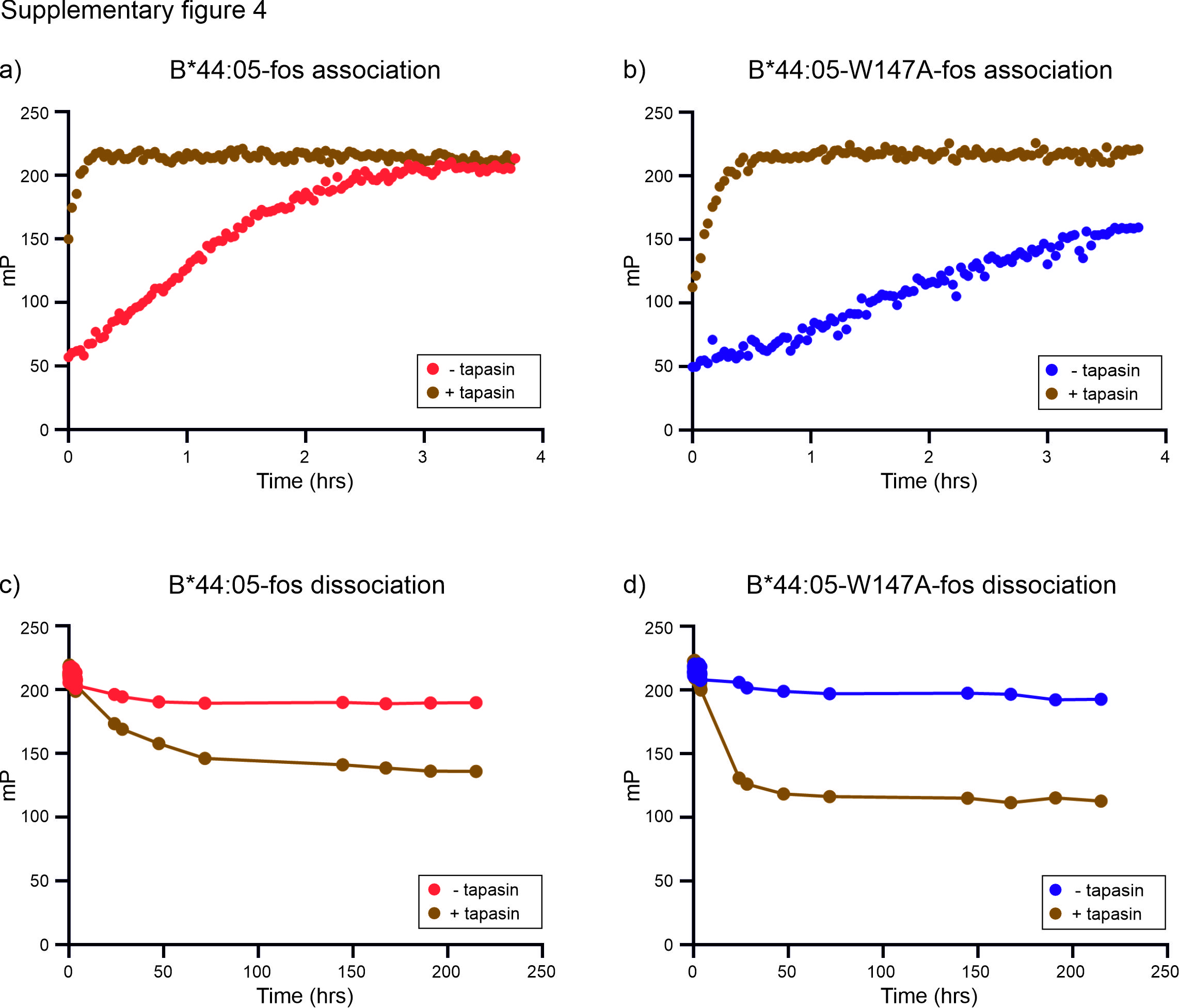

Supplement: Supplementary Figure 4 — Peptide binding and peptide dissociation experiments with HLA-B*44:05-fos and HLA-B*44:05-W147A-fos in the presence or absence of tapasin-jun-ERp57. (a) Peptide binding experiment in which 160 nM HLA-B*44:05-fos was supplemented with 3.2 µM β2-microglobulin and UV exposed, before being added to 2 nM EEFGK*AFSF peptide in the presence or absence of 300 nM tapasin-jun-ERp57. Fluorescence polarization was measured at 25°C. (b) Peptide binding experiment with HLA-B*44:05-W147A-fos as in Supplementary Figure 4a . (c) Peptide dissociation experiment in which 160 nM HLA-B*44:05-fos was supplemented with 3.2 µM β2-microglobulin, UV exposed and incubated overnight with 2 nM EEFGK*AFSF peptide. The next day, 66.7 µM EENLLDFVRF peptide competitor was added in the presence or absence of 300 nM tapasin-jun-ERp57. Fluorescence polarization was measured at 25°C. (d) Peptide dissociation experiment with HLA-B*44:05-W147A-fos as in Supplementary Figure 4c . [file Image4.jpeg]

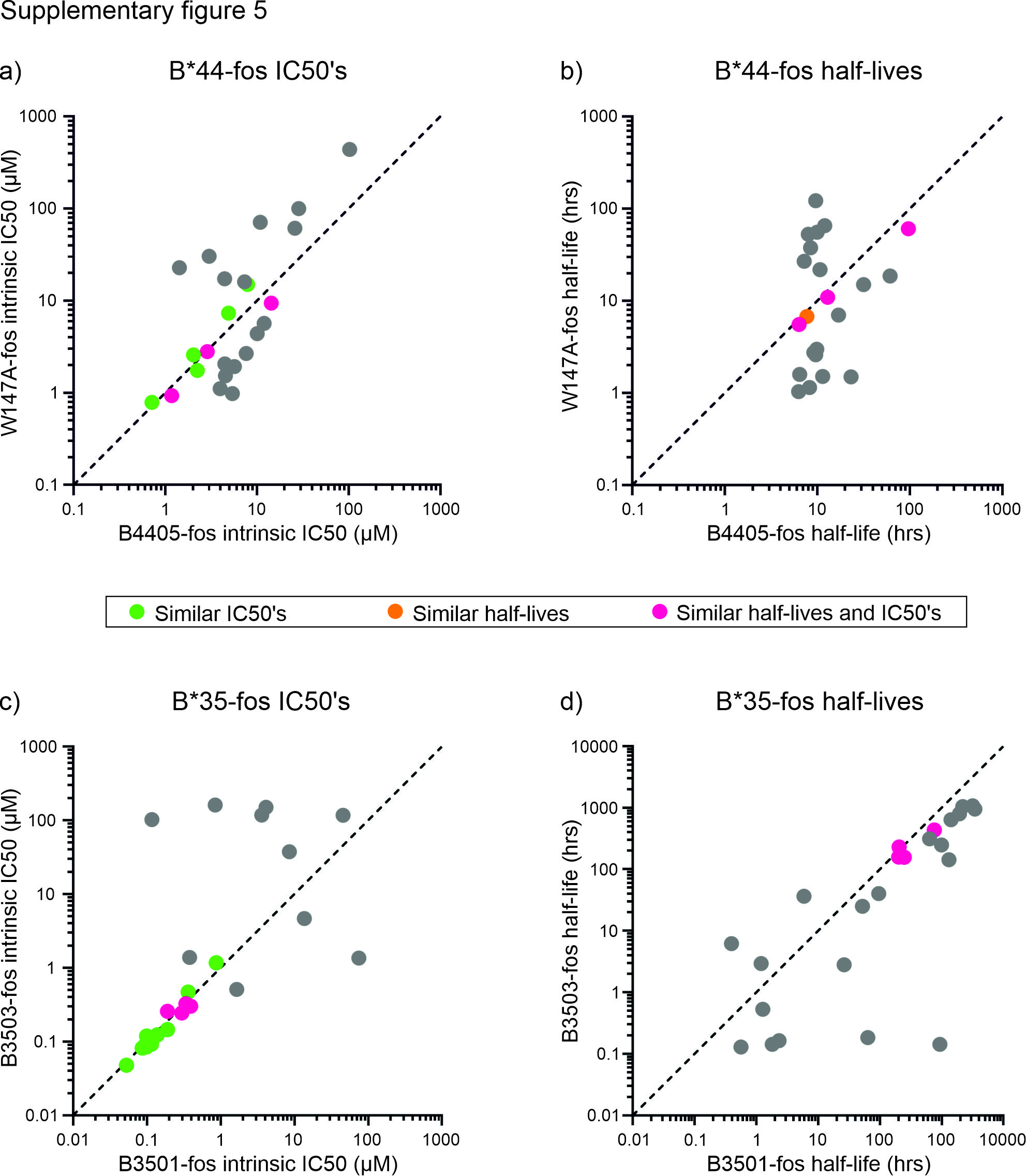

Supplement: Supplementary Figure 5 — Identification of similar binding peptides. (a) The mean intrinsic IC50 value of each peptide competing for binding to HLA-B*44:05-fos is shown on the x-axis, while the mean intrinsic IC50 value measured for each peptide competing for binding to HLA-B*44:05-W147A-fos is shown on the y-axis. To identify peptides with similar abilities to compete for binding to both HLA-B*44:05-fos and HLA-B*44:05-W147A-fos, the ratio by which the intrinsic IC50s differed was calculated. Peptides with an intrinsic IC50 ratio of less than 2 are shown in green or shown in pink if the peptides also have a half-life ratio of less than 2, all other peptides are shown in grey. (b) The mean half-lives of the peptide-HLA-B*44:05-fos complexes are shown on the x-axis, while the mean half-lives of the peptide-HLA-B*44:05-W147A-fos complexes are shown on the y-axis. To identify peptides that formed complexes with HLA-B*44:05-fos and HLA-B*44:05-W147A-fos with similar half-lives, the ratio by which the half-lives differed was calculated. Peptides with a half-life ratio of less than 2 are shown in orange or shown in pink if the peptides also have an intrinsic IC50 ratio of less than 2, all other peptides are shown in grey. (c) The mean intrinsic IC50 value of each peptide competing for binding to HLA-B*35:01-fos is shown on the x-axis, while the mean intrinsic IC50 value measured for each peptide competing for binding to HLA-B*35:03-fos is shown on the y-axis. Peptides with similar intrinsic IC50 ratios were calculated as in Supplementary Figure 5a . (d) The mean half-lives of the peptide-HLA-B*35:01-fos complexes are shown on the x-axis, while the mean half-lives of the peptide-HLA-B*35:03-fos complexes are shown on the y-axis. Peptides with similar half-life ratios were calculated as in Supplementary Figure 5b . [file Image5.jpeg]

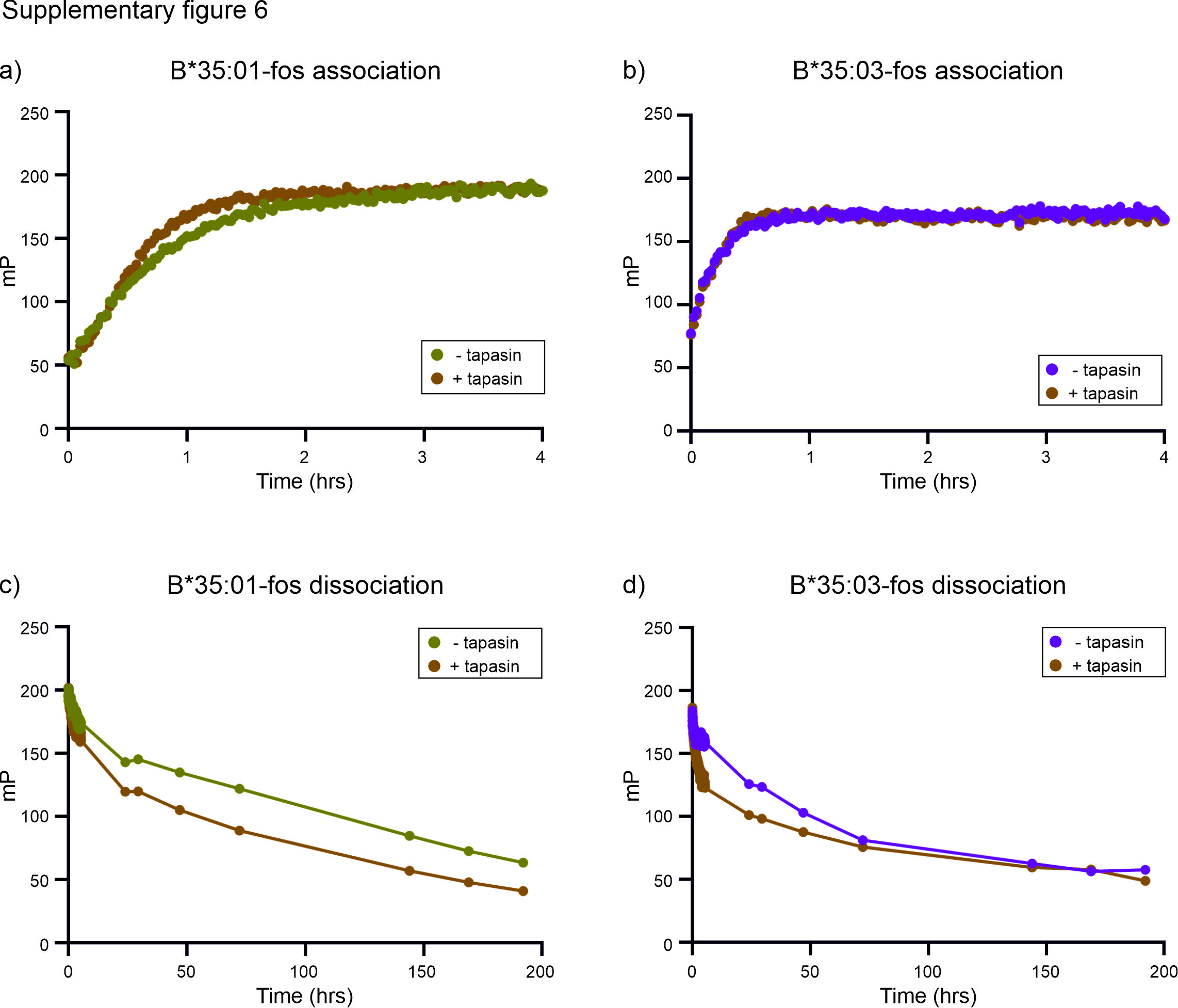

Supplement: Supplementary Figure 6 — Peptide binding and peptide dissociation experiments with HLA-B*35:01-fos and HLA-B*35:03-fos in the presence or absence of tapasin-jun-ERp57. (a) Peptide binding experiment in which 225 nM HLA-B*35:01-fos was supplemented with 4.5 µM β2-microglobulin and UV exposed, before being added to 3 nM YPLK*EQHGM peptide in the presence or absence of 300 nM tapasin-jun-ERp57. Fluorescence polarization was measured at 25°C. (b) Peptide binding experiment with HLA-B*35:03-fos as in Supplementary Figure 6a . (c) Peptide dissociation experiment in which 225 nM HLA-B*35:01-fos was supplemented with 4.5 µM β2- microglobulin and UV exposed and incubated overnight with 3 nM YPLK*EQHGM peptide. The next day, 66.7 µM YPLHEQHGM peptide competitor was added in the presence or absence of 300 nM tapasin-jun-ERp57. Fluorescence polarization was measured at 25°C. (d) Peptide dissociation experiment with HLA-B*35:03-fos as in Supplementary Figure 6c . [file Image6.jpeg]

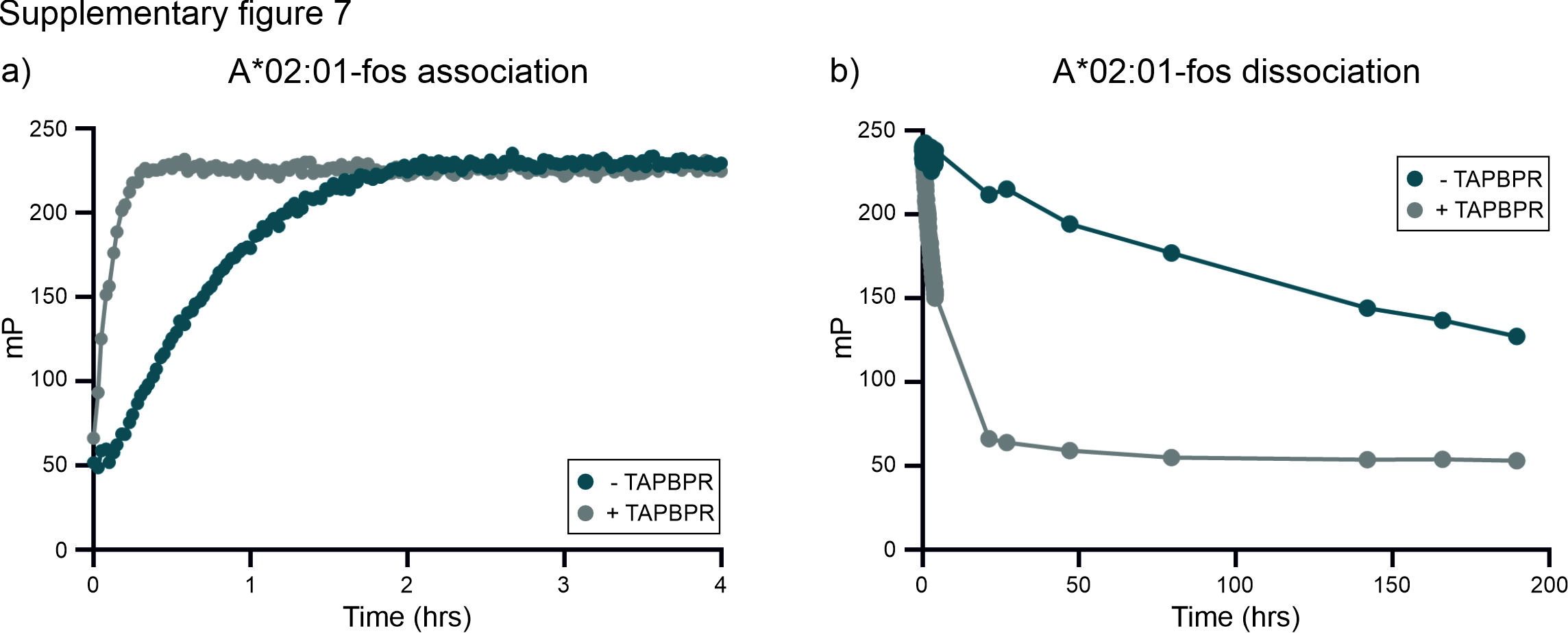

Supplement: Supplementary Figure 7 — Peptide binding and peptide dissociation experiments with HLA-A*02:01-fos in the presence or absence of TAPBPR. (a) Peptide binding experiment in which 50 nM HLA-A*02:01-fos was supplemented with 1 µM β2-microglobulin and UV exposed, before being added to 2 nM FLPSDC*FPSV peptide in the presence or absence of 300 nM TAPBPR. Fluorescence polarization was measured at 25°C. (b) Peptide dissociation experiment in which 50 nM HLA-A*02:01-fos was supplemented with 1 µM β2-microglobulin and UV exposed and incubated overnight with 2 nM FLPSDC*FPSV peptide. The next day, 66.7 µM FLPSDCFPSV peptide competitor was added in the presence or absence of 300 nM TAPBPR and fluorescence polarization was measured at 25°C. [file Image7.jpeg]
